# Evaluation of Beta-Lactamase-Producing Strains Isolated in a Tertiary Nephrology Hospital in Romania

**DOI:** 10.3390/antibiotics15060580

**Published:** 2026-06-07

**Authors:** Edgar-Costin Chelaru, Andrei-Alexandru Muntean, Ioana Manea, Mihai-Octav Hogea, Crina-Mihaela Dinuță, Mioara Mazăre, Mădălina-Maria Muntean, Călin-Constantin Ghițulescu, Bogdan-Florin Ciomaga, Costin-Ștefan Caracoti, Diana-Maria Preoteasa, Mircea Ioan Popa

**Affiliations:** 1 Discipline of Microbiology II, Department 2, Faculty of Medicine, Carol Davila University of Medicine and Pharmacy, 020021 Bucharest, Romania; alexandru.muntean@umfcd.ro (A.-A.M.); ioana.manea@drd.umfcd.ro (I.M.); octav.hogea@umfcd.ro (M.-O.H.); crina-mihaela.dinuta@rez.umfcd.ro (C.-M.D.); mioara.mazare@drd.umfcd.ro (M.M.); madalina.muntean@umfcd.ro (M.-M.M.); calin-constantin.ghitulescu@drd.umfcd.ro (C.-C.G.); bogdan-florin.ciomaga@drd.umfcd.ro (B.-F.C.); costin-stefan.caracoti@drd.umfcd.ro (C.-Ș.C.); diana-maria.preoteasa@rez.umfcd.ro (D.-M.P.); 2 Cantacuzino National Military Medical Institute for Research and Development, 050096 Bucharest, Romania; 3 Dr. Carol Davila Clinical Nephrology Hospital, 010731 Bucharest, Romania

**Keywords:** beta-lactam resistance, carbapenemase, Gram-negative bacteria

## Abstract

**Background/Objectives:** Given the ongoing threat of antimicrobial resistance, the identification and characterization of multidrug-resistant isolates are essential. An increase in antimicrobial-resistant bacteria has been reported in Romania, but national data are still scarce. This study aimed to evaluate beta-lactamase-producing Gram-negative bacteria (GNB) isolated over two years at a Romanian nephrology hospital, while comparing carbapenemase detection phenotypic methods. **Methods**: Gram-negative bacterial isolates collected between January 2022 and May 2024 that met antimicrobial resistance screening criteria were evaluated. After identification, extensive disk diffusion antibiograms were performed, read, and interpreted, complemented by testing on cloxacillin/oxacillin-supplemented Mueller–Hinton agar. The colistin minimum inhibitory concentration (MIC) was not assessed, and aztreonam–avibactam was not tested for Enterobacterales. For non-fermenter GNB, the colistin MIC was determined. Phenotypic carbapenemase production tests were performed for all strains (BlueCarba Test, CIM, mCIM, zCIM, and rCIM). Carbapenemase detection immunochromatographic tests were performed for a set of strains. **Results**: Among the 397 evaluated strains, 335 (84.38%) were Enterobacterales and 62 (15.62%) non-fermenter GNB, showing high antimicrobial resistance levels. Of these, 188 (47.35%) were *Klebsiella pneumoniae*; 139/188 (73.93%) showed carbapenem resistance and carbapenemase production; 49/188 (26.06%) produced two carbapenemases; and 45/188 (23.93%) presented resistance to all tested antimicrobials. MALDI-TOF identified 28 KPC-producing *K. pneumoniae* strains. Lateral flow assays revealed NDM, VIM, KPC, and OXA-48-like enzymes in 48 of 56 tested Enterobacterales; 12/48 strains produced two carbapenemases. Of the 62 non-fermenter GNB, 33 were *Pseudomonas* spp. and 20 *Acinetobacter baumannii*; one *Pseudomonas* spp. was susceptible only to colistin and seven only to cefiderocol; four *A. baumannii* were susceptible only to colistin and three only to cefiderocol. Lateral flow assays detected VIM or IMP enzymes in 13/33 *Pseudomonas* spp. and OXA-23 and/or OXA-40/-58 enzymes in all 20 *A. baumannii*. **Conclusions**: Among the evaluated strains, many showed resistance to multiple antimicrobial classes. Furthermore, strains co-producing two carbapenemases were identified.

## 1. Introduction

Antimicrobial resistance (AMR) is a worldwide phenomenon with a severe impact on public health. It is estimated that AMR causes more than 1 million deaths annually. In 2019, AMR was directly responsible for 1.27 million deaths, and it contributed to 4.95 million deaths globally [1]. The selection of bacteria with reduced susceptibility to bactericidal and bacteriostatic substances is caused by exposure to improper treatment regimens, failure to comply with hygiene rules, and inadequate measures to prevent their spread in the environment by healthcare providers, but also by the food industry. This can lead to the emergence of multidrug-resistant (MDR) bacterial strains. Such sources can become difficult to control and can be further responsible for severe, hard-to-treat, or impossible-to-treat infections. Of particular concern are patients with chronic kidney disease and those managed with urological devices such as urinary catheters, nephrostomies, or JJ stents, who represent a high-risk population for urinary tract infections caused by MDR microorganisms and for whom comorbidities and altered organ function constrain the therapeutic options [1,2,3,4,5,6,7,8,9,10,11].

Although high levels of antimicrobial resistance have been reported in Romania and progress has been made in combating this phenomenon (antibiotic stewardship programs, guidelines, medical staff training, antibiotic consumption reduction measures, surveillance and screening for MDR carriage, etc.), the currently available data remain insufficient for robust national-level statistical extrapolation [12,13,14,15,16,17,18,19]. For these reasons, continuing and increasing efforts to identify, report, and combat this phenomenon are needed.

Among the most common resistance mechanisms, both in terms of spread and impact on public health, are enzymatic mechanisms. An important group is represented by beta-lactamases, which include extended-spectrum beta-lactamases (ESBLs), cephalosporinases (e.g., AmpCs), and carbapenemases. Carbapenemases can inactivate most beta-lactams by hydrolysis. The genes encoding such enzymes are frequently located on mobile genetic elements (e.g., plasmids), which facilitate horizontal transmission and environmental spread. Not only are healthcare facilities affected, but MDR microorganisms and their resistance mechanisms can also be identified in the community. Beta-lactamases are frequently associated with Gram-negative bacteria (GNB), found either among Enterobacterales or in non-fermenters such as *Pseudomonas* spp. and *Acinetobacter* spp. An important classification system for these enzymes is the Ambler classification [20,21,22,23,24,25,26,27,28,29,30].

It is well known that carbapenemases, which are classified as Ambler classes A, B, and D, can hydrolyze broad-spectrum antibiotics such as carbapenems. Although an important number of carbapenemases have been described, five of them are the most common, also known as the “Big Five”: KPC (Class A); NDM, VIM, and IMP (Class B, metallo-beta-lactamases or MBLs); and OXA-48/OXA-48-like (Class D). Carbapenemases confer high resistance to beta-lactams, including aztreonam for classes A and D and novel beta-lactamase inhibitors for class B (e.g., avibactam). The resources available for treating infections caused by bacteria harboring such enzymatic equipment are limited, sometimes resulting in therapeutic failure and negative consequences for patients and the healthcare system [8,20,21,31,32,33].

When resistance mechanisms against multiple antimicrobial classes are associated, extended-drug-resistant (XDR) and pan-drug-resistant (PDR) bacteria can emerge. Such microorganisms may need unconventional treatment regimens or may even lack therapeutic options [34,35,36,37,38].

The presence of various resistance mechanisms can be established by phenotypic methods. These methods include the interpretative reading of the disk diffusion antibiogram (including reduced diameters and/or resistance to certain “key” antibiotics); various tests including combination disk testing (CDT), the double-disk synergy test (DDST), colorimetric tests (e.g., Carba NP, BlueCarba Test), and the carbapenem inactivation method (CIM) and its derivatives (mCIM, modified CIM; zCIM, zinc CIM (developed for better MBL producer identification, which are zinc-dependent); rCIM-A, rapid CIM-AmpC (developed for excluding AmpC false positives), etc.), as well as lateral flow assays (e.g., NG-Test^®^ CARBA 5, O.K.N.V.I. RESIST-5 Coris BioConcept, RESIST-ACINETO Coris BioConcept), etc. Molecular methods, such as the polymerase chain reaction (PCR) and whole-genome sequencing (WGS), can be used to identify resistance genes. Other methods, such as the detection of carbapenem hydrolysis by matrix-assisted laser desorption/ionization time-of-flight mass spectrometry (MALDI-TOF MS), have also been described, and, more recently, the possibility of identifying carbapenemases directly with MALDI-TOF was introduced, particularly for KPC [19,39,40,41,42,43,44,45,46].

The objective of this study was to extensively evaluate and characterize beta-lactamase-producing Gram-negative bacteria (GNB) isolated over two years at a Romanian nephrology hospital using phenotypic methods and to provide further evidence regarding antimicrobial resistance in our country. The overall antimicrobial susceptibility of these strains and their phenotypic resistance were evaluated, with a particular focus on beta-lactamases. Moreover, the multiple carbapenemase detection phenotypic assays were evaluated and their performance across methods was compared, given their known specific limitations.

## 2. Results

The 397 isolated strains were identified and grouped as Enterobacterales (*n* = 335, 84.38%) and non-fermenter GNB (*n* = 62, 15.62%). The Enterobacterales were identified as 3 (0.75%) *Citrobacter* spp. (1 *C. braakii*, 2 *C. freundii*); 10 (2.5%) *Enterobacter* spp. (1 *E. asburiae*, 1 *E. bugandensis*, 4 *E. cloacae*, 1 *E. hormaechei*, 2 *E. kobei*, 1 *E. xiangfangensis*); 83 (20.9%) *Escherichia coli*; 1 (0.25%) *Hafnia alvei*; 193 (48.61%) *Klebsiella* spp. (2 *K. aerogenes*, 3 *K. oxytoca*, 188 *K. pneumoniae*); 6 (1.51%) *Morganella morganii*; 11 (2.77%) *Proteus mirabilis*; 14 (3.5%) *Providencia* spp. (5 *P. rettgeri*, 9 *P. stuartii*); 1 (0.25%) *Raoultella ornithinolytica*; and 13 (3.2%) *Serratia* spp. (7 *S. marcescens*, 6 *S. ureilytica*). The non-fermenter GNB were identified as 2 (0.5%) *Achromobacter xylosoxidans*; 20 (5.03%) *Acinetobacter baumannii*; 1 (0.25%) *Burkholderia cepacia*; 4 (1%) *Myroides odoratimimus*; 32 (8.06%) *Pseudomonas aeruginosa*; 1 (0.25%) *Pseudomonas monteilii*; and 2 (0.5%) *Stenotrophomonas maltophilia* (Table 1).

The Enterobacterales strains were initially isolated from bile (*n* = 1), percutaneous endoscopic gastrostomy (PEG) tubes (*n* = 1), ascites fluid (*n* = 2), pleural fluid (*n* = 4), intravenous catheters (*n* = 9), blood/blood cultures (*n* = 15), purulent and wound secretions (*n* = 21), lower respiratory tract secretions (*n* = 28), and urine (*n* = 254, including one from both urine and blood) collected by a spontaneous urination/urinary catheter, nephrostomy, or JJ stent. The non-fermenter GNB strains were isolated from pleural fluid (*n* = 2), blood/blood cultures (*n* = 5), lower respiratory tract secretions (*n* = 13), purulent and wound secretions (*n* = 14), and urine (*n* = 28) (Table 2).

### 2.1. Enterobacterales

#### 2.1.1. Antimicrobial Susceptibility of Enterobacterales

Of the total of 335 Enterobacterales isolates, 92.84% (*n* = 311) showed resistance to ampicillin; 90.15% (*n* = 302) to ticarcillin; 90.45% (*n* = 303) to piperacillin; 71.04% (*n* = 238) to amoxicillin–clavulanic acid; 65.07% (*n* = 218) to ticarcillin–clavulanic acid; 64.18% (*n* = 215) to piperacillin–tazobactam; 50.45% (*n* = 169) to mecillinam; 43.28% (*n* = 145) to temocillin; 56.12% (*n* = 188) to cefoxitin; 78.21% (*n* = 262) to cefotaxime; 74.93% (*n* = 251) to ceftazidime; 74.03% (*n* = 248) to cefepime; 45.97% (*n* = 154) to ertapenem; 38.51% (*n* = 129) to meropenem; 37.91% (*n* = 127) to imipenem; 68.06% (*n* = 228) to aztreonam; 26.57% (*n* = 89) to ceftazidime–avibactam; 51.04% (*n* = 171) to ceftolozane–tazobactam; 33.13% (*n =* 111) to cefiderocol; 75.52% (*n* = 253) to ciprofloxacin; 74.63% (*n* = 250) to levofloxacin; 62.09% (*n* = 208) to tobramycin; 52.54% (*n* = 176) to gentamicin; 46.57% (*n* = 156) to amikacin; 62.09% (*n* = 208) to trimethoprim–sulfamethoxazole; 39.40% (*n* = 132) to chloramphenicol; 0.3% (*n* = 1) to tigecycline; 1.49% (*n* = 5) to fosfomycin–trometamol; and 0.3% (*n* = 1) to nitrofurantoin.

**Figure 1:**
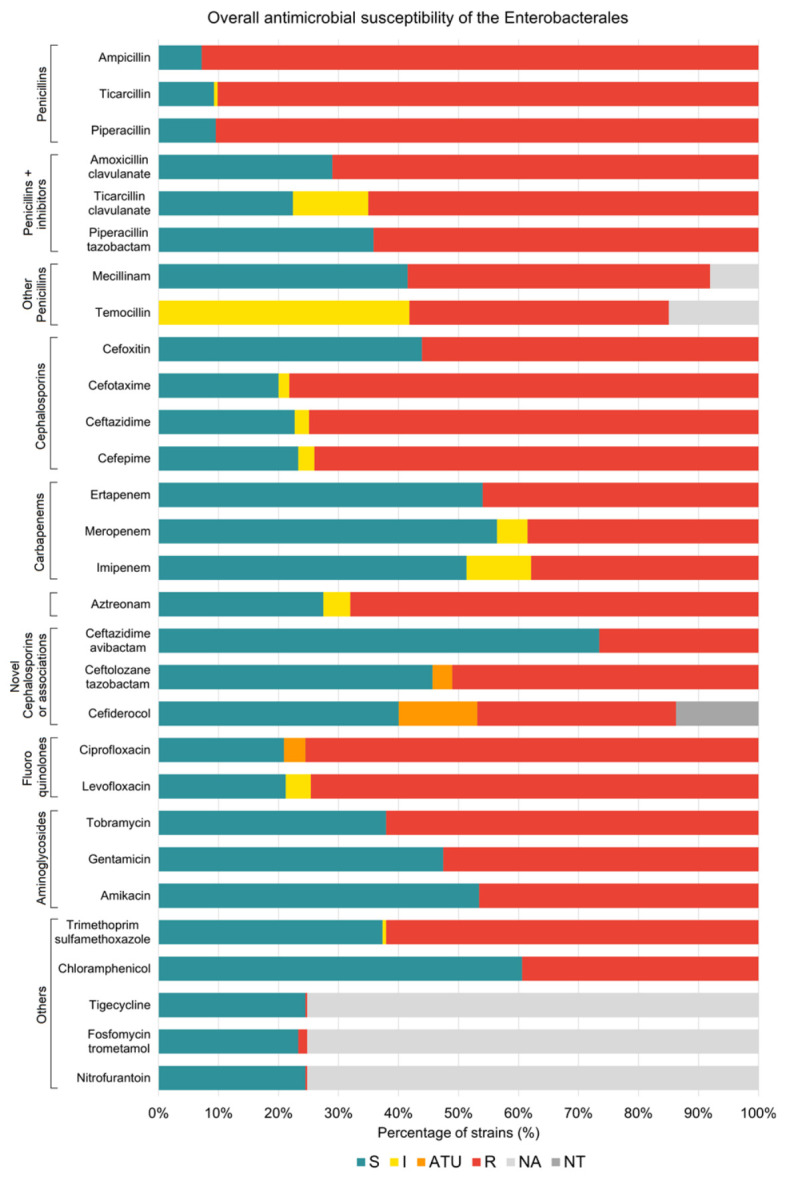
Overall antimicrobial susceptibility of the 335 Enterobacterales isolates evaluated in the study. S = susceptible; I = susceptible, increased exposure; R = resistant; ATU = area of technical uncertainty; NA = not applicable; NT = not tested. Note: Amoxicillin–clavulanate susceptibility data only apply to intravenous administration. Tigecycline susceptibility data only apply to *E. coli* and *C. koseri* (*n* = 83/335 *E. coli*; no *C. koseri* in the study). Susceptibility data only apply for urinary tract infection (UTI) treatment for temocillin (*E. coli*, *Klebsiella* spp., other than *K. aerogenes*, and *P. mirabilis*, *n* = 285/335); nitrofurantoin (*E. coli*, *n* = 83/335); fosfomycin–trometamol (*E. coli*, *n* = 83/335), intravenous administration; and mecillinam (*E. coli*, *Citrobacter* spp., *Klebsiella* spp., *Raoultella* spp., *Enterobacter* spp., and *P. mirabilis*, *n* = 308/335), oral administration.

Excluding strains for which cefiderocol was not available (*n =* 46), 38.40% (*n* = 111/289) of the tested strains could be reported as resistant to cefiderocol. Excluding the strains for which testing was not applicable and therefore not performed, 50.88% (*n* = 145/285) showed resistance to temocillin; 54.87% (*n* = 169/308) to mecillinam; 1.2% (*n* = 1/83) to tigecycline; 6.02% (*n* = 5/83) to fosfomycin–trometamol; and 1.2% (*n* = 1/83) to nitrofurantoin. A detailed figure showing the antimicrobial susceptibility for all Enterobacterales isolates is provided (Figure 1).

#### 2.1.2. Phenotypic Beta-Lactam Resistance Mechanisms of Enterobacterales

**Figure 2:**
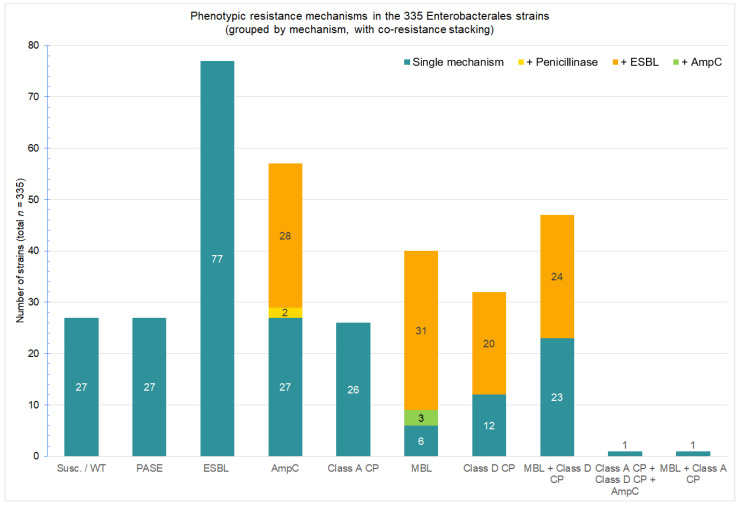
Phenotypic resistance mechanisms observed on the disk diffusion antibiogram for the 335 Enterobacterales strains, grouped by mechanism, with associated resistance stacking. Susc./WT = susceptible/wild type; PASE = penicillinase; ESBL = extended-spectrum beta-lactamase; AmpC = cephalosporinase; CP = carbapenemase producer; MBL = metallo-beta-lactamase.

The phenotypic resistance mechanisms identified by reading and interpreting the disk diffusion antibiograms for the 335 Enterobacterales isolates revealed 147 (43.88%) carbapenemase producer (CP) strains: 26 Class A CPs (e.g., KPC) associated with non-enzymatic resistance mechanisms (e.g., efflux pumps, impermeability, etc.); 40 Class B CPs (MBLs), with 3/40 associated with an AmpC and 31/40 associated with an ESBL; 32 Class D CPs (e.g., OXA-48-like), with 20/32 associated with an ESBL; 47 MBL + Class D carbapenemase co-producers, with 24/47 associated with an ESBL; 1 MBL + Class A carbapenemase co-producer; and 1 Class A + Class D carbapenemase co-producer, associated with an AmpC. The remaining 188 (56.12%) Enterobacterales strains were identified as 57 AmpC producers (28/57 co-produced an ESBL and 2/57 co-produced a penicillinase); 77 ESBL producers; 27 penicillinase producers; and 27 strains showing an overall susceptible phenotype. It must be noted that variations in susceptibility were observed, likely due to differences among strains, including the presence of non-enzymatic-associated resistance mechanisms (efflux pumps, impermeability, etc.) (Figure 2).

**Figure 3:**
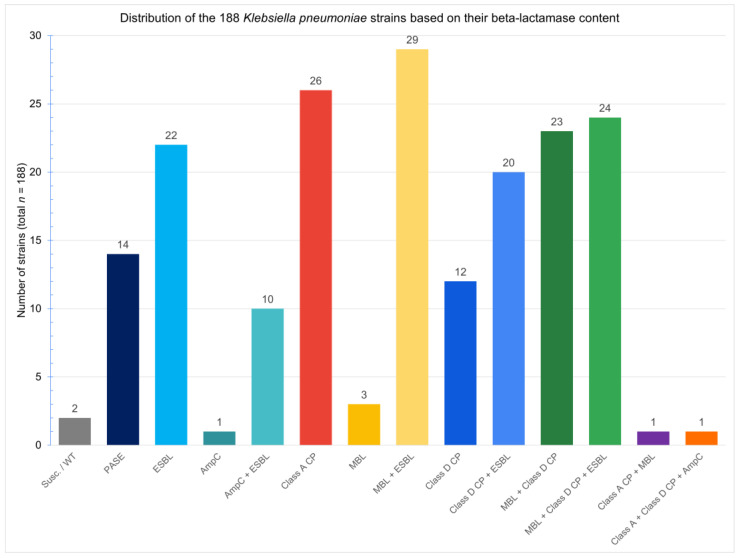
Distribution of the 188 *Klebsiella pneumoniae* strains based on their phenotypically identified beta-lactamase content. Susc./WT = susceptible/wild type; PASE = penicillinase; ESBL = extended-spectrum beta-lactamase; AmpC = cephalosporinase; CP = carbapenemase producer; MBL = metallo-beta-lactamase.

Among the evaluated Enterobacterales isolates, 188/335 (56.12%) strains were *K. pneumoniae*. Of these, 139/188 (73.93%) crossed the threshold for carbapenemase screening and tested positive for carbapenemase production on the disk diffusion antibiogram. All 26 Class A CPs, all 32 Class D CPs, 32/40 MBL producers, and all 49 strains co-producing two carbapenemases (47 MBL + Class D, 1 MBL + Class A, and 1 Class A + Class D) reported above were strains of *K. pneumoniae*. These strains co-harbored ESBLs, AmpCs, or other beta-lactam resistance mechanisms (Figure 3).

It must be noted that 45/188 (23.93%) strains of *Klebsiella pneumoniae* were resistant to all antimicrobials included in the disk diffusion panel; as minimum inhibitory concentrations (MICs) were not determined for colistin and ceftazidime–avibactam, and aztreonam–avibactam was not tested at all, formal XDR/PDR classification cannot be confirmed. Phenotypically, these strains were 1 Class A CP; 1 Class D CP + ESBL producer; 1 MBL producer; 1 MBL + Class A CP; 9 MBL + Class D CPs; 18 MBL + Class D CP + ESBL producers; and 14 MBL + ESBL producers (Table 3).

The eight remaining MBL producers, as identified phenotypically on the disk diffusion antibiogram, were three strains of *P. stuartii*, two strains of *S. ureilytica*, one strain of *E. hormaechei*, one strain of *P. mirabilis,* and one strain of *E. coli*.

**Figure 4:**
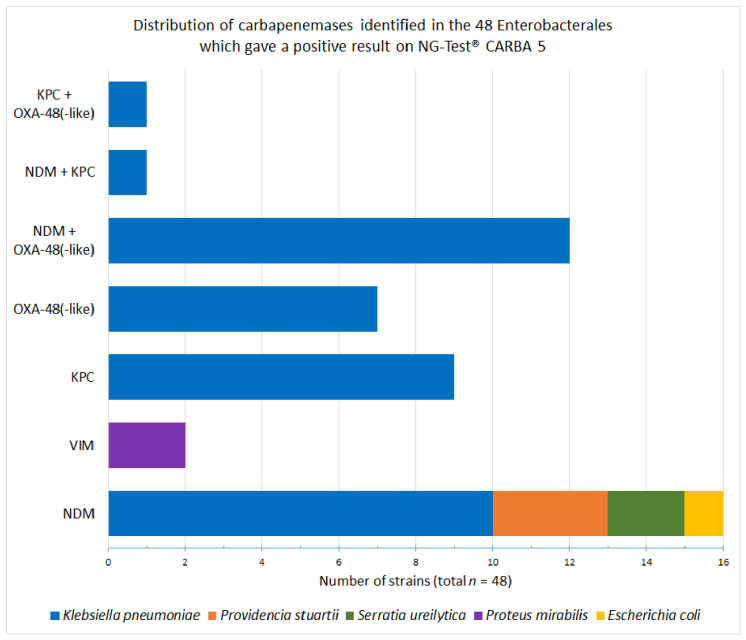
Distribution of carbapenemases identified in the 48 Enterobacterales showing a positive result on NG-Test^®^ CARBA 5.

The carbapenemases identified immunochromatographically in 48 Enterobacterales strains (40 *K. pneumoniae*, 3 *P. stuartii*, 2 *S. ureilytica*, 2 *P. mirabilis*, and 1 *E. coli*) were NDM (*n* = 29), VIM (*n* = 2), KPC (*n* = 11), and/or OXA-48-like (*n* = 20); 12 of the total tested strains co-produced NDM + OXA-48, one strain NDM + KPC, and one strain KPC + OXA-48. The microorganisms and carbapenemases were associated as follows: the three *P. stuartii*, two *S. ureilytica*, and one *E. coli* strains were NDM producers; the two *P. mirabilis* were VIM producers. Of the 40 CP *K. pneumoniae*, 9 were KPC producers; 10 were NDM producers; 7 were OXA-48-like producers; 12 were NDM + OXA-48-like producers; 1 was an NDM + KPC producer; and 1 was a KPC + OXA-48-like producer (Figure 4).

For eight more strains for which the NG-Test^®^ CARBA 5 was performed, no carbapenemase was detected. Phenotypically (on the disk diffusion antibiogram), they were identified as one AmpC + ESBL-producing *K. pneumoniae*; one AmpC-producing *K. pneumoniae*; one natural, inducible AmpC-producing *P. stuartii*; one AmpC hyper-producing *E. coli*; one natural, inducible AmpC-producing *S. marcescens*; one natural, inducible AmpC-producing *E. kobei*; one non-CTX-M ESBL-producing *K. pneumoniae*; and one MBL-producing *P. mirabilis*.

MALDI-TOF MS directly identified KPC in 28/188 *K. pneumoniae* strains. However, three strains that were not identified by MALDI-TOF as KPC-producing isolates tested positive for KPC production on the NG-Test^®^ CARBA 5 and had an antibiogram that was phenotypically highly suggestive of a Class A carbapenemase. A fourth strain, with a positive phenotype for Class A carbapenemase production, was also not identified as a KPC producer by MALDI-TOF.

The BlueCarba test (BCT) was positive for 137/335 strains, with 88 showing an early positive result for CPs (before the 2 h incubation period). An inconclusive result was obtained in 16/335 strains due to either a weak reaction (+) or a positive reaction in the negative control. CIM revealed 137/335 CP strains; zCIM 147/335 CP strains; mCIM 144/335 CP strains; and rCIM-A 151/335 CP strains. Overall, 172/335 strains were detected as non-CPs by all six assays (the five carbapenemase-producing assays and the disk diffusion antibiogram), while 122/335 were detected as CPs by all six assays. Excluding the BCT’s inconclusive results, 179/335 strains were detected as non-CPs by all assays, while 129/335 were detected as CPs by all assays. Furthermore, 6/335 tested negative on 5/6 assays, and 1/335 tested negative on 4/6 assays; 12/335 tested positive on 5/6 assays, and 5/335 tested positive on 4/6 assays. Cohen’s *κ* (kappa) inter-rater agreement coefficients were calculated for the results obtained after evaluating the disk diffusion antibiogram for carbapenemase production (DD-AST CP), BCT, CIM, zCIM, mCIM, and rCIM-A, with the results shown in Table 4.

Of the strains showing carbapenemase production on the NG-Test^®^ CARBA 5, 37/48 were positive on all six assays, and 5/48 were positive on 5/6 assays. The inter-rater agreement was also calculated for the 56 strains, considering the positive/negative carbapenemase-producing result of the NG-Test^®^ CARBA 5 performed, in relation to the phenotypic tests (Table 5).

Due to the kappa paradox, low kappa values were obtained for the 56 strains tested with the NG-Test^®^ CARBA 5, despite small differences (e.g., a one-point difference between zCIM and NG-Test^®^ CARBA 5 resulted in a kappa value of 0.93). This was caused by a high prevalence of positive results, as Cohen’s kappa is sensitive to imbalances in outcome prevalence (48/56 positive on NG-Test^®^ CARBA; 49/56 positive on rCIM-A; 47/56 positive on DD-AST and zCIM; 45/56 positive on mCIM and BCT; and 40/56 positive on CIM). To aid the interpretation of the agreement results, the percentage agreement was also calculated for the 56 strains, and the results are shown in Table 6.

### 2.2. Non-Fermenters

#### 2.2.1. Antimicrobial Susceptibility of Non-Fermenter GNB

**Figure 5:**
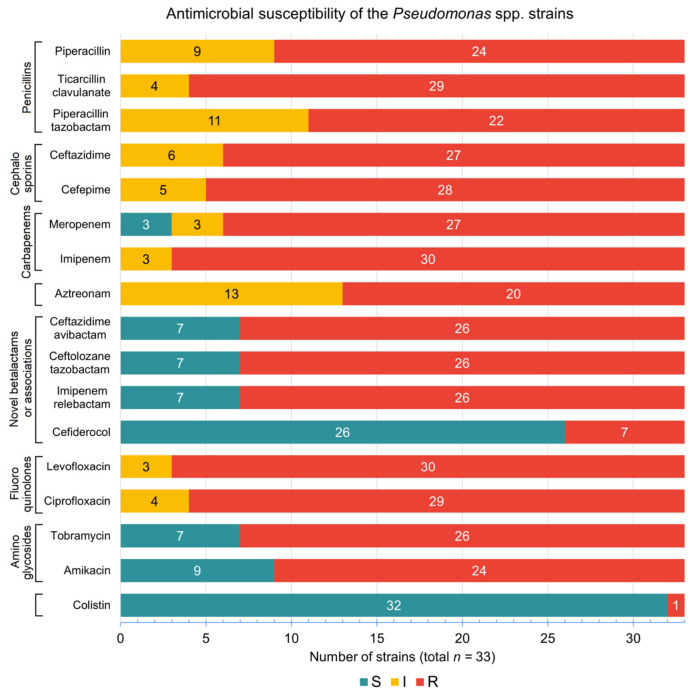
Antimicrobial susceptibility of the 33 *Pseudomonas* spp. strains (32 *Pseudomonas aeruginosa* and 1 *Pseudomonas monteilii*). S = susceptible; I = susceptible, increased exposure; R = resistant.

The 33 strains of *Pseudomonas* spp. showed resistance to the tested antimicrobials as follows: 24/33 to piperacillin, 29/33 to ticarcillin–clavulanic acid, 22/33 to piperacillin–tazobactam, 27/33 to ceftazidime, 28/33 to cefepime, 27/33 to meropenem, 30/33 to imipenem, 20/33 to aztreonam, 26/33 to ceftazidime–avibactam, 26/33 to ceftolozane–tazobactam, 26/33 to imipenem–relebactam, 7/33 to cefiderocol, 30/33 to levofloxacin, 29/33 to ciprofloxacin, 26/33 to tobramycin, 24/33 to amikacin, and 1/33 to colistin (Figure 5 and Table 7). It should be noted that the colistin-resistant strain was susceptible to cefiderocol, so none of the *Pseudomonas* strains were resistant to all antimicrobials in the tested panel.

**Figure 6:**
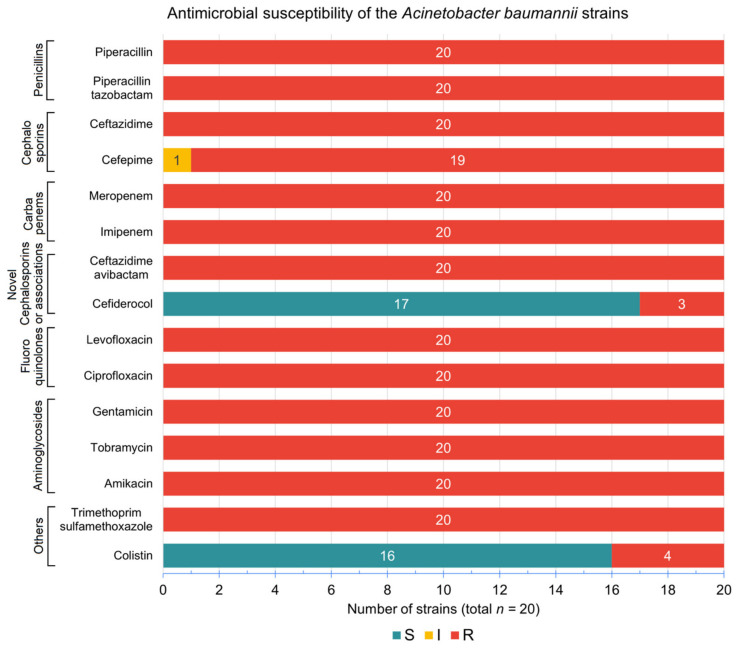
Antimicrobial susceptibility of the 20 *Acinetobacter baumannii* strains. S = susceptible; I = susceptible, increased exposure; R = resistant.

The 20 *Acinetobacter baumannii* strains showed resistance to the tested antimicrobials as follows: all 20 strains were resistant to piperacillin, piperacillin–tazobactam, ceftazidime, ceftazidime–avibactam, imipenem, meropenem, levofloxacin, ciprofloxacin, gentamicin, tobramycin, amikacin, and trimethoprim–sulfamethoxazole; 19/20 strains were resistant to cefepime; 3/20 strains were resistant to cefiderocol; and 4/20 strains were resistant to colistin (Figure 6 and Table 8). It should be noted that the three strains showing resistance to cefiderocol were susceptible to colistin, so none of the *A. baumannii* strains were resistant to all antimicrobials in the tested panel.

Both *Achromobacter xylosoxidans* strains were susceptible to meropenem, trimethoprim–sulfamethoxazole, and piperacillin–tazobactam. Both *Stenotrophomonas maltophilia* strains were susceptible to levofloxacin and minocycline and susceptible with increased exposure to trimethoprim–sulfamethoxazole. The *Burkholderia cepacia* strain was susceptible to ceftazidime, meropenem, minocycline, and trimethoprim–sulfamethoxazole.

Similarly to Enterobacterales, the non-fermenter GNB strains presented variations in susceptibility due to associated resistance mechanisms.

#### 2.2.2. Phenotypic Beta-Lactam Resistance Mechanisms of Non-Fermenter GNB

The interpretation of the disk diffusion antibiogram for *Pseudomonas* spp. revealed 13/33 MBL producers, associated with AmpC and/or ESBL and/or non-enzymatic mechanisms (e.g., impermeability, porin loss, efflux pumps, etc.); 15/33 AmpC, AmpC + ESBL, or ESBL producers; 2/33 carbapenem-resistant through non-enzymatic mechanisms; and 3/33 overall susceptible/wild-type strains. The disk diffusion antibiogram was highly suggestive of the presence of Class D carbapenemases (e.g., OXA-23/-58) in all 20 *A. baumannii* isolates, in conjunction with an ESBL and/or an AmpC and/or other non-enzymatic resistance mechanisms.

**Figure 7:**
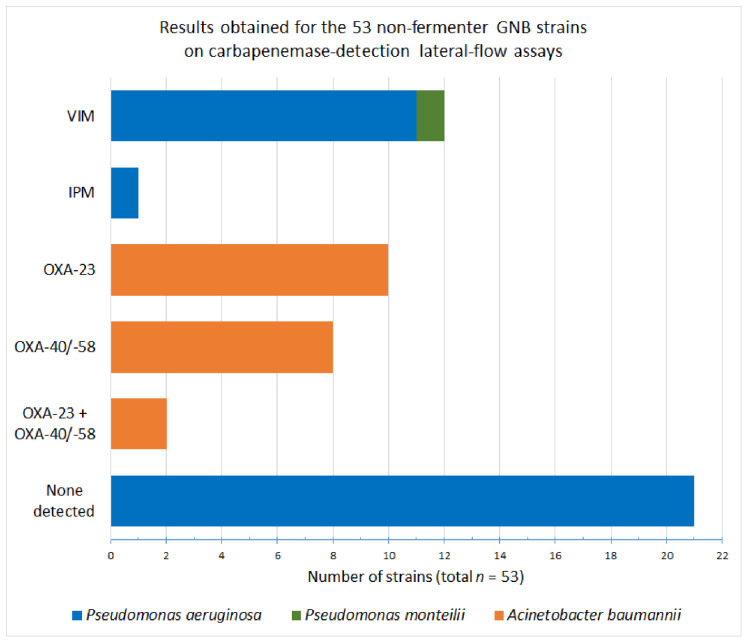
Results obtained for the 53 non-fermenter Gram-negative bacterial strains (33 *Pseudomonas* spp. and 20 *Acinetobacter baumannii*) on carbapenemase detection lateral flow assays.

Carbapenemase detection lateral flow assays performed for the 33 *Pseudomonas* spp. strains revealed 1 IMP-producing *P. aeruginosa* and 12 VIM-producing *Pseudomonas* spp. (11 *P. aeruginosa* and 1 *P. monteilii*). All 20 *Acinetobacter baumannii* strains were detected as carbapenemase producers on lateral flow assays: 10 OXA-23-producing strains; 8 OXA-40/-58-producing strains; and 2 OXA-23 + OXA-40/-58-producing strains. The results obtained on the carbapenemase detection lateral flow assays for non-fermenter GNB are presented in Figure 7.

The BlueCarba test (BCT) was positive in 16/33 *Pseudomonas* spp. and 14/20 *Acinetobacter baumannii* and inconclusive in 2/20 *Acinetobacter baumannii*; 10/16 BCT-positive *Pseudomonas* spp. strains gave an early positive result (before incubation), and all 10 were MBL producers. CIM was positive for 11/20 *A. baumannii* and 10/33 *Pseudomonas* spp.; zCIM for 13/20 *A. baumannii* and 14/33 *Pseudomonas* spp.; mCIM for 20/20 *A. baumannii* and 14/33 *Pseudomonas* spp.; and rCIM-A for 20/20 *A. baumannii* and 13/33 *Pseudomonas* spp.

Cohen’s *κ* (kappa) inter-rater agreement coefficients were calculated for the results of the carbapenemase detection assays performed for the 53 non-fermenter GNB strains, with the results shown in Table 9.

Detailed data and results for the evaluated strains are provided in the Supplementary Table (Table S1).

## 3. Discussion

As mentioned above, one strain of *P. mirabilis*, suspected phenotypically to produce MBLs, tested negative for carbapenemase production on the NG-Test^®^ CARBA 5. For 4/48 for which a positive result for carbapenemase production was obtained on the NG-Test^®^ CARBA 5, the disk diffusion antibiogram did not offer a clear conclusion: two KPC-producing *K. pneumoniae* were identified as Class D (e.g., OXA-48-like) + ESBL producers; one NDM + KPC-producing *K. pneumoniae* was identified as an MBL + Class D (e.g., OXA-48-like) producer; and one NDM + OXA-48-like-producing *K. pneumoniae* was identified as a Class D (OXA-48-like) + ESBL producer. For two strains of *P. mirabilis* showing an AmpC phenotype on the antibiogram (including susceptibility to carbapenems and ceftazidime–avibactam), zCIM, mCIM, and rCIM-A gave positive results. Due to these inconsistencies, the NG-Test^®^ CARBA 5 was performed, and the two strains were identified as VIM producers; a similar pattern was observed for a third strain. Carbapenemases showing reduced hydrolytic activity and highly zinc-dependent MBLs may be associated with such phenomena [21,32]. Furthermore, several discordances were observed for all carbapenemase-producing assays on Enterobacterales: zCIM presented a higher positivity rate in strains with an MBL phenotype compared to BCT and CIM; BCT presented positive or inconclusive results in several strains detected as non-CP by the other assays; rCIM-A presented overall consistent results compared to the antibiogram, zCIM, mCIM, and NG-Test^®^ CARBA 5, but we must note that three non-CP strains (according to the other assays) were detected as positive (the strain profile was as follows: one penicillinase-producing *K. pneumoniae*, one non-CTX-M ESBL-producing *K. pneumoniae,* and one AmpC-producing *S. marcescens*). These strains presented very mucoid or adherent growth on culture media; strain-related factors, other than carbapenemase production, may influence the performance of these assays.

It is important to note that MALDI-TOF MS did not detect a KPC in 4/32 strains of *K. pneumoniae,* for which phenotypic tests were highly suggestive of a KPC (three detected immunochromatographically), but a KPC was found in a *K. pneumoniae* phenotypically suspected to be an MBL + ESBL producer. For these strains, molecular evaluation by PCR or next-generation/whole-genome sequencing (NGS/WGS) is necessary. This may help to determine whether the results obtained from the disk diffusion antibiogram evaluation were false positives, whether the strain produced a carbapenemase that could not be detected by lateral flow assays, or whether a KPC variant could not be identified by MALDI-TOF MS, as more than 150 *bla*_KPC_ gene variants have been reported worldwide, with many new variants reported since 2019. It has been noted that *K. pneumoniae* producing KPC-2 or -3 variants are susceptible to mutations that are not easily recognized clinically and may be missed. Mutational structural changes have led to the occurrence of variants, including avibactam-resistant KPCs (e.g., KPC-31 and -33, mutants derived from KPC-2 and KPC-3). Such mutants, in addition to their modified susceptibility profiles, were reported to give false negative results in lateral flow assays such as the NG-Test^®^ CARBA 5 (e.g., *bla*_KPC-31_, *bla*_KPC-33_, *bla*_KPC-76_). *K. pneumoniae* producing KPC-2 and -3 variants were reported in Europe (e.g., Greece, Italy), isolated from various clinical samples, including rectal swabs for the KPC-33 variant [47]. Moreover, false negative and false positive results were reported for KPC detection by MALDI-TOF [42].

The evaluation of the antibiogram revealed further interesting suspicions, including the presence of non-CTX-M-type ESBLs and Class D, OXA-type ESBLs; the presence of an ACC-type AmpC; the presence of penicillinases resistant to beta-lactamase inhibitors; strains suspected of OXA-181 or OXA-163 production, etc. Such observations could be extremely valuable in selecting strains that could require molecular evaluation.

In the case of non-fermenter GNB, it must be mentioned that several strains of *Pseudomonas aeruginosa* showed carbapenem resistance and an overall difficult-to-treat profile, but most probably not through carbapenemase production. As no carbapenemase was detected on the lateral flow assays, and the carbapenemase detection method results were negative, the resistance is most likely caused by non-enzymatic mechanisms such as impermeability, efflux pumps, porin loss, or mutations. However, we must acknowledge that, due to the absence of molecular evaluation, we cannot fully confirm the findings or exclude the possibility that certain carbapenemases, which may not be detected by lateral flow assays or phenotypic carbapenemase detection methods, could be encoded and/or produced by these strains.

A curious phenomenon was the occurrence of eight rough colony-producing *Klebsiella pneumoniae* (as seen in Figure S1). These strains are associated with high levels of resistance to antibiotics and were phenotypically as follows: three MBL + Class D (e.g., OXA-48-like) producers and five MBL + Class D (OXA-48-like) + ESBL producers. Of these eight strains, four were detected by lateral flow assays as NDM + OXA-48-like producers. In a study published by Wyres et al., similar phenotypes were reported in strains associated with alterations in capsular polysaccharide biosynthesis (e.g., disruption of *wzi/wza* genes) and/or lipopolysaccharide truncation, which can paradoxically enhance biofilm formation and complement resistance. Rough morphotypes have also been reported in high-risk *K. pneumoniae* sequence types (e.g., ST258, ST512) and may serve as a rapid visual indicator for high-resistance strains [48]. The strains identified in our study should be further investigated both phenotypically, by studying the structural characteristics of the microorganisms and by stress-inducing capsule formation through inoculation on selective media (such as MacConkey), and by molecular analysis (such as WGS).

MIC determination through broth dilution methods was not performed for Enterobacterales in this study. As disk diffusion antimicrobial susceptibility testing for colistin is no longer recommended, the data obtained may be unreliable. However, testing using a 10 µg colistin disk was performed, following previous guidelines for historical/exploratory reference; the obtained data are summarized in Figure S2 [49].

Among the evaluated isolates, 27 strains showed a susceptible or even wild-type profile, so the strain selection process might have been partially biased—some strains were sent for evaluation, although screening should have been negative. It must be mentioned, however, that some of these strains were isolated from patients with severe infections and that the microorganisms manifested a hyper-virulent profile and/or a very mucoid phenotype; these aspects might have influenced the decision to send them for extensive evaluation. Moreover, we cannot exclude the possibility that resistant strains isolated during the selection period were not sent for evaluation.

It is extremely concerning that 45 *Klebsiella pneumoniae* strains showed resistance to all antimicrobials in the tested panel. It should be noted that, for 5/45, cefiderocol susceptibility was not tested. For these strains, MICs should be determined by broth microdilution (for both colistin and ceftazidime–avibactam), as well as aztreonam–avibactam (AZA) susceptibility testing, in order to establish if the strains fall under the extended-drug-resistant (XDR) or pan-drug-resistant (PDR) categories [38].

Given the high number of carbapenemases identified, and the fact that beta-lactamase-encoding genes are frequently located on plasmids and other conjugative genetic elements, concerns regarding the dissemination of such genes must be raised. The horizontal transfer of genes such as *bla*_KPC_, *bla*_NDM_, and *bla*_OXA-48_ has been documented, especially among microorganisms involved in hospital-acquired infections, leading to the emergence of multidrug-resistant microorganisms that may be associated with multiple resistance mechanisms and, clinically, may lead to therapeutic failure. Furthermore, it has been reported that such genes can spread silently, amplifying the phenomenon, as such microorganisms can go undetected [6,7,50,51,52].

Further evaluation of the strains by studying their structures using the Bruker Daltonics Biotyper and their molecular profiles by PCR and/or whole-genome next-generation sequencing, combining short and long reads, is considered, as it would provide epidemiologically valuable information.

### Limitations

Several limitations must be acknowledged. First, the selection of the bacterial strains was not based on a systematic sampling strategy; strains were selected based on clinical suspicion and screening criteria, which may have introduced selection bias. Second, the episodic nature of the collection over January 2022–May 2024 prevented temporal trend analysis. Third, patient-level demographic and clinical data were not collected and analyzed for this study; extrapolation to population-level incidence or prevalence was therefore not appropriate. Fourth, colistin and ceftazidime–avibactam MICs were not determined, and aztreonam–avibactam susceptibility was not tested for Enterobacterales due to a lack of resources. Moreover, cefiderocol was only available for 289/335 Enterobacterales strains; susceptibility testing for these antimicrobials is considered for future work. Fifth, no molecular method (e.g., PCR, WGS) was available for detecting and comparing resistance genes; further molecular evaluation is planned. Sixth, antimicrobial classes other than beta-lactams were not explored in depth; this limitation will be addressed during future molecular evaluation.

## 4. Materials and Methods

### 4.1. Isolate Selection and Identification

A total of 397 bacterial strains, isolated between January 2022 and May 2024 in the Dr. Carol Davila Clinical Hospital of Nephrology, Bucharest, Romania, were selected and evaluated for antimicrobial resistance in the Cantacuzino National Military Medical Institute for Research and Development, Bucharest, Romania. The microorganisms were isolated from clinical specimens in the hospital laboratory, and the strains that were resistant to beta-lactams and suspected of carbapenemase production were sent to our microbiology laboratory. The EUCAST (European Committee on Antimicrobial Susceptibility Testing) positive screening criteria for carbapenemase production were generally followed (disk diffusion diameter < 28 mm for meropenem and/or <25 mm for ertapenem, in the case of Enterobacterales), but strains showing reduced diameters for ceftazidime–avibactam and/or ceftolozane–tazobactam were also considered [19,53]. The strains were initially isolated from bile (*n* = 1), PEG tubes (*n* = 1), ascites fluid (*n* = 2), pleural fluid (*n* = 6), intravenous catheters (*n* = 9), blood/blood cultures (*n* = 20), purulent and wound secretions (*n* = 35), lower respiratory tract secretions (*n* = 41), and urine collected spontaneously/through urinary catheters, via nephrostomy or via a JJ stent (*n* = 282, including 1 from both urine and blood) (summarized in Table 1 and Table 2).

The bacterial strains were subcultured on chromogenic agar (UriSelect™4 Medium, BioRad, Marne-la-Coquette, France) to assess culture purity. This culture medium was chosen as it is supplemented with chromogenic enzymatic substrates, which allow the identification of mixed cultures, since various microorganisms produce pigmented colonies (as detailed in the manufacturer’s insert). Moreover, this culture medium allowed the growth of all microorganisms evaluated in this study [54].

Identification/species confirmation was performed with MALDI-TOF mass spectrometry (Bruker Daltonics, Bremen, Germany). Isolated colonies from pure cultures were picked with the tips of sterile bacteriological loops and applied to the wells of the MALDI-TOF target plate. Using an Eppendorf Research Plus 1–10 µL micropipette (Eppendorf, Hamburg, Germany), 1 µL of freshly prepared Bruker Daltonics matrix (α-cyano-4-hydroxycinnamic acid, HCCA) was applied over each culture-containing well. All identifications were performed in triplicate; if scores ≥ 2.00 were not obtained, the reading process was repeated. As the Bruker Daltonics MALDI-TOF MS MBT Compass HT IVD software (v.5.2.300) and database allowed the detection of KPC in *Klebsiella pneumoniae*, the presence of such enzymes was also assessed during identification [42].

### 4.2. Antimicrobial Susceptibility Testing

Extensive disk diffusion antibiograms (Kirby–Bauer) were performed following the protocols outlined in the EUCAST guidelines, complemented by information from CA-SFM (Comité de l’antibiogramme de la Société Française de Microbiologie). For all isolates, 0.5 McFarland suspensions were prepared in sterile saline water; a V-1 plus vortex was used to prepare the suspensions, and a calibrated Den-1B densitometer was used for turbidity measurement (Biosan, Riga, Latvia). The suspensions were inoculated with sterile swabs onto Mueller–Hinton agar (MHA, Oxoid, Basingstoke, UK) within 15 min of preparation. Oxoid antimicrobial disks were applied using antibiotic dispensers (Oxoid, Basingstoke, UK), following the 15 min rule. As the guidelines recommend, certain antimicrobials were used either to assess susceptibility in certain species or for screening purposes, as noted below. *Escherichia coli* ATCC 25922 was used as a quality control (QC) strain for disk diffusion antibiograms (to evaluate sterility, antimicrobial activity, and culture media) [19,32,55,56,57,58].

The Enterobacterales isolates were tested with amikacin (AK 30 μg), ampicillin (AMP 10 μg), amoxicillin–clavulanic acid (AMC 20–10 μg), aztreonam (ATM 30 μg), cefepime (FEP 30 μg), cefiderocol (FDC 30 μg, available for 289 of the total Enterobacterales), cefotaxime (CTX 5 μg), cefoxitin (FOX 30 μg, for AmpC screening), ceftazidime (CAZ 10 μg), ceftazidime–avibactam (CZA 10–4 μg), ceftolozane–tazobactam (C/T 30–10 μg), chloramphenicol (C 30 μg), ciprofloxacin (CIP 5 μg), colistin (CT 10 μg; we emphasize that this practice is normally discouraged), ertapenem (ETP 10 μg), fosfomycin–trometamol (FF 200 μg, for *E. coli*), gentamicin (CN 10 μg), imipenem (IPM 10 μg), levofloxacin (LEV 5 μg), mecillinam (MEC 10 μg, for *E. coli, Citrobacter* spp., *Klebsiella* spp., *Raoultella* spp., *Enterobacter* spp., and *P. mirabilis*), meropenem (MEM 10 μg), netilmicin (NET 10 μg, for screening), nitrofurantoin (F 100 μg, for *E. coli*), piperacillin (PRL 30 μg), piperacillin–tazobactam (TZP 30–6 μg), temocillin (TEM 30 μg, for *E. coli*, *Klebsiella* non-*aerogenes*, *P. mirabilis*; used with all strains for OXA-48 screening), ticarcillin (TIC 75 μg), ticarcillin–clavulanic acid (TCC 75–10 μg), tigecycline (TGC 15 μg, for *E. coli* and *C. koseri*), tobramycin (TOB 10 μg), and trimethoprim–sulfamethoxazole (SXT 1.25–23.75 μg) [19,32,53,55,56,57,58].

The *Pseudomonas* spp. were tested by disk diffusion antibiograms with amikacin (AK 30 μg), aztreonam (ATM 30 μg), cefepime (FEP 30 μg), cefiderocol (FDC 30 μg), ceftazidime (CAZ 10 μg), ceftazidime–avibactam (CZA 10–4 μg), ciprofloxacin (CIP 5 μg), imipenem (IPM 10 μg), imipenem–relebactam (I/R 10–25 μg), levofloxacin (LEV 5 μg), meropenem (MEM 10 μg), piperacillin (PRL 30 μg), piperacillin–tazobactam (TZP 36 μg), ticarcillin–clavulanic acid (TCC 75–10 μg), and tobramycin (TOB 10 μg) [19,53,57,58].

Susceptibility to colistin was established by evaluating the minimum inhibitory concentration (MIC). Broth microdilutions were performed using Thermo Scientific™ Sensititre FRCOL AST plates (ThermoFisher Scientific, Waltham, MA, USA). For each strain, a 0.5 McFarland suspension was prepared in demineralized water using a calibrated Sensititre™ nephelometer (Thermo Fisher Scientific, Waltham, MA, USA). With an Eppendorf Research Plus 1–10 µL micropipette (Eppendorf, Hamburg, Germany), 10 µL of suspension was added to a Sensititre™ cation-adjusted Mueller–Hinton broth tube (Thermo Fisher Scientific, Waltham, MA, USA) to obtain the final suspension. Using a Sensititre AIM™ automated inoculation delivery system, 50 µL of the Mueller–Hinton broth suspension was inoculated into each well of one row of the Thermo Scientific™ Sensititre FRCOL AST plates (colistin concentrations ranging from 0.12 μg/mL to 128 μg/mL). Each plate was used to test 6 strains + 2 controls. *E. coli* ATCC 25922 was used as a negative control, and *E. coli* NCTC 13846, a colistin-resistant strain exhibiting the colistin resistance gene *mcr*-1 (mobilized colistin resistance-1), was used as a positive control. The plates were incubated for 24 h at 37 °C. Reading was performed visually by two investigators using a Sensititre™ manual viewbox (Thermo Fisher Scientific, Waltham, MA, USA) [19,53,57,58].

The *Acinetobacter baumannii* strains were tested by disk diffusion antibiograms with amikacin (AK 30 μg), cefepime (FEP 30 μg), cefiderocol (FDC 50 μg), ceftazidime (CAZ 30 μg), ceftazidime–avibactam (CZA 30–20 μg), ciprofloxacin (CIP 5 μg), imipenem (IPM 10 μg), levofloxacin (LEV 5 μg), meropenem (MEM 10 μg), piperacillin (PRL 30 μg), piperacillin–tazobactam (TZP 110 μg), tobramycin (TOB 10 μg), gentamicin (CN 10 μg), and trimethoprim–sulfamethoxazole (SXT 1.25–23.75 μg). The different concentrations for CAZ, CZA, PRL, and TZP were chosen as suggested by CA-SFM and interpreted accordingly. Similarly, as performed for *Pseudomonas* spp., susceptibility to colistin was determined by evaluating the MIC: broth microdilutions were performed using Thermo Scientific™ Sensititre FRCOL AST plates (Thermo Fisher Scientific, Waltham, MA, USA), as described above for *Pseudomonas* spp. [19,53,57,58].

Additionally, as suggested in CA-SFM, *Achromobacter xylosoxidans* was tested with meropenem (MEM 10 μg), piperacillin–tazobactam (TZP 36 μg), and trimethoprim–sulfamethoxazole (SXT 1.25–23.75 μg); *Burkholderia cepacia* with ceftazidime (CAZ 10 μg), meropenem (MEM 10 μg), minocycline (MH 30 μg), and trimethoprim–sulfamethoxazole (SXT 1.25–23.75 μg); and *Stenotrophomonas maltophilia* with levofloxacin (LEV 5 μg), minocycline (MH 30 μg), and trimethoprim–sulfamethoxazole (SXT 1.25–23.75 μg), as they are intrinsically resistant to carbapenems due to an endogenous metallo-beta-lactamase. *Achromobacter xylosoxidans, Burkholderia cepacia, Stenotrophomonas maltophilia*, and *Myroides odoratimimus* were included as they were selected via the same screening workflow. For *Myroides odoratimimus*, no EUCAST or CA-SFM breakpoints are available; susceptibility data for this species are therefore not reported [19,53,57,58].

The plates were incubated for 18–24 h at 35–37 °C, and reading was performed using the ADAGIO automated system (BioRad, Marne-la-Coquette, France) and interpreted according to the EUCAST 2025 breakpoint tables (v.15). For ticarcillin interpretation, the EUCAST 2022 breakpoint tables (v.12) were used, and, for the particular cases mentioned above, the CA-SFM guidelines were used. Disk diffusion inhibition diameters for *Escherichia coli* ATCC 25922 were read and compared with the EUCAST QC tables [19,53,57,58].

### 4.3. Phenotypic Beta-Lactamase Production Assessment

In-house-prepared MHA (Oxoid, Basingstoke, UK) supplemented with 300 mg/L oxacillin (Oxacilina^®^ 500 mg/1000 mg injectable powder, Antibiotice, Iași, Romania) (MHO300) for Enterobacterales, and MHA supplemented with 1000 mg/L or 2000 mg/L cloxacillin (Orbenin^®^ 1 g injectable powder, Astellas Pharma, Levallois Perret, France) (MHC1000 and MHC2000) for *Acinetobacter* and *Pseudomonas*, was prepared and used in order to assess the presence of cephalosporinases (AmpCs) and underlying Class A ESBLs, as previously described [59,60,61,62,63,64,65]. The non-fermenters were tested on MHC with all antibiotics; the Enterobacterales were tested on MHO only with aztreonam (ATM 30 μg), cefotaxime (CTX 5 μg), cefoxitin (FOX 30 μg), ceftazidime (CAZ 10 μg), ertapenem (ETP 10 μg), ticarcillin (TIC 75 μg), and ticarcillin–clavulanic acid (TCC 75–10 μg).

The interpretative reading of the plates was performed as previously described [19,32,53,55,56]. For Enterobacterales, resistance to penicillins only was marked as penicillinase. High suspicion regarding ESBLs (Class A or D) was raised by resistance to beta-lactams up to third-generation cephalosporins, to monobactams, and sometimes to ertapenem; resistance up to fourth-generation cephalosporins is, however, common for CTX-M-type ESBLs (Class A) [66]. Inhibition by beta-lactamase inhibitors (including the positive double-disk synergy test, DDST, between cefotaxime and/or ceftazidime and clavulanic acid) was marked as indicating the presence of a Class A ESBL (e.g., CTX-M). Although resistance to cefoxitin is an important marker of AmpCs, certain cephalosporinases, such as ACC-1, lack hydrolytic activity against cefoxitin, making testing on Mueller–Hinton supplemented with cloxacillin or oxacillin useful in this regard. As such, an increase of at least 5 mm in the inhibition diameter around the antimicrobial disk placed on MHO300 (or MHC1000/2000 for non-fermenters) compared to MHA indicated the presence of an AmpC [19,53,55,56,57,58,67,68].

For Enterobacterales strains, carbapenemase production was evaluated phenotypically by assessing resistance to key beta-lactam agents. Resistance to carbapenems and aztreonam, with susceptibility (or at least a large diameter) to ceftazidime–avibactam, together with a diameter > 6 mm around temocillin, was marked as indicating the presence of a Class A carbapenemase (e.g., KPC). Resistance to carbapenems and to ceftazidime–avibactam but susceptibility to Aztreonam was marked as indicating the presence of a Class B carbapenemase (metallo-beta-lactamase, MBL). Resistance to carbapenems, with susceptibility to/a large diameter for ceftazidime–avibactam and a diameter of 6 mm around temocillin, was marked as indicating the presence of a Class D carbapenemase (an OXA-type carbapenemase pertaining to the OXA-48-like group). The presence of other associated resistance mechanisms, such as ESBLs, efflux pumps, and porins, sometimes led to aztreonam resistance in MBL producers or to decreased diameters around ceftazidime–avibactam in the case of serine-carbapenemase producers; such situations were interpreted accordingly. Similar criteria were applied for non-fermenter GNB; additionally, for *Pseudomonas* spp., increased diameters around the imipenem–relebactam disk compared to the imipenem disk were suggestive of an AmpC or a Class A carbapenemase (further distinguished on MHC2000), while a decreased or no diameter was suggestive of MBL production. A decreased diameter around carbapenems, with large diameters around other beta-lactams, was suggestive of non-enzymatic mechanisms such as impermeability, efflux pumps, or porin loss (e.g., OprD mutations) [19,32,53,55,56,69].

In order to phenotypically assess carbapenemase production, the Blue Carba test (BCT) [70,71], CIM [39,72], mCIM [39,73,74,75], zCIM [43], and rCIM-A [44,45] were also performed for all strains, as described in the published protocols, using Oxoid broth, MHA, and antibiotics (Oxoid, Basingstoke, UK), Eppendorf Research Plus micropipettes and consumables (Eppendorf, Hamburg, Germany), a V-1 plus vortex, a V-32 Multi-Vortex, a Microspin 12 Plus High-speed Mini-centrifuge, and a calibrated Den-1B densitometer (Biosan, Riga, Latvia) when necessary. When no special protocol developed for non-fermenter GNB was available, the protocol published for Enterobacterales was applied. It should be noted that, in this study, the CLSI mCIM protocol was used, rather than the improved protocol recently developed by Simner et al. [76]. Additionally, lateral flow assays were performed for selected Enterobacterales strains (NG-Test^®^ CARBA 5, NG Biotech Laboratories, Guipry-Messac, France); for all *Pseudomonas* spp. strains (O.K.N.V.I. RESIST-5 Coris BioConcept, Gembloux, Belgium); and for all *Acinetobacter baumannii* strains (RESIST-ACINETO Coris BioConcept, Gembloux, Belgium) in order to detect carbapenemase production, if the enzyme was covered by the test [40,41].

### 4.4. Data Analysis

Statistical analysis and chart creation were performed using Microsoft Office Excel v.16.0. For statistical analysis, the Excel Data Analysis ToolPak add-in was used.

Descriptive statistics were used to summarize data on isolates and antimicrobial resistance. Cohen’s *κ* (kappa) inter-rater agreement coefficients were calculated to compare the results obtained for the carbapenemase production assays. The kappa results were interpreted as follows: < 0, no agreement; 0–0.20, slight (poor) agreement; 0.21–0.40, fair agreement; 0.41–0.60, moderate agreement; 0.61–0.80, substantial (good) agreement; 0.81–1, almost perfect (very good) agreement. Because Cohen’s kappa is sensitive to imbalances in outcome prevalence, particularly when one outcome category predominates (the kappa paradox), the percentage agreement was additionally calculated for the strains tested using the NG-Test^®^ CARBA 5 to aid the interpretation of the agreement results. The 95% confidence intervals (CI) were calculated for all results.

References were managed using Zotero v.6.0.36.

## 5. Conclusions

Many of the evaluated strains show alarming levels of antimicrobial resistance, including to carbapenems. Carbapenemase production is frequently associated with these strains.

Although no statistically relevant data on incidence and prevalence can be reported, significant variability in carbapenemases and highly resistant strains co-producing two carbapenemases were identified.

The interpretative reading of the disk diffusion antibiogram remains a valuable, cheap, fast, and useful method, not only for the phenotypic evaluation of antimicrobial susceptibility but also for detecting and discriminating among resistance mechanisms. Certain carbapenemases may be overlooked due to their weak activity or high zinc requirements.

Phenotypic assays for detecting carbapenemase production can detect the presence of such enzymes in Enterobacterales with higher reliability than in non-fermenter Gram-negative bacteria, but all of them show limitations, especially in the presence of weak and/or highly zinc-dependent carbapenemases. For this reason, applying molecular assays is necessary to obtain highly reliable data.

For the phenotypic diagnosis of carbapenemase-producing Gram-negative bacterial strains, antibiotic susceptibility testing and carbapenemase detection assays should be complementary, as certain strains may be overlooked or falsely identified as carbapenemase producers when evaluating the antibiogram, while others might remain undetected by carbapenemase production assays.

## Figures and Tables

**Table 1:** Data on the 397 identified and evaluated isolates.

Group	Number and Percentage of Isolates per Group	Number (*n*) and Percentage (%) of Isolates per Genus and Species
Enterobacterales	335 (84.38%)	*Citrobacter* spp. (*n* = 3) (0.75%) - *C. braakii* (*n* = 1) (0.25%) - *C. freundii* (*n* = 2) (0.5%) *Enterobacter* spp. (*n* = 10) (2.5%) - *E. asburiae* (*n* = 1) (0.25%) - *E. bugandensis* (*n* = 1) (0.25%) - *E. cloacae* (*n* = 4) (1%) - *E. hormaechei* (*n* = 1) (0.25%) - *E. kobei* (*n* = 2) (0.5%) - *E. xiangfangensis* (*n* = 1) (0.25%) *Escherichia coli* (*n* = 83) (20.9%) *Hafnia alvei* (*n* = 1) (0.25%) *Klebsiella* spp. (*n* = 193) (48.61%) - *K. aerogenes* (*n* = 2) (0.5%) - *K. oxytoca* (*n* = 3) (0.75%) - *K. pneumoniae* (*n* = 188) (47.35%) *Morganella morganii* (*n* = 6) (1.51%) *Proteus mirabilis* (*n* = 11) (2.77%) *Providencia* spp. (*n* = 14) (3.5%) - *P. rettgeri* (*n* = 5) (1.26%) - *P. stuartii* (*n* = 9) (2.26%) *Raoultella ornithinolytica* (*n* = 1) (0.25%) *Serratia* spp. (*n* = 13) (3.2%) - *S. marcescens* (*n* = 7) (1.76%) - *S. ureilytica* (*n* = 6) (1.51%)
Non-fermenter Gram-negative bacteria	62 (15.62%)	*Achromobacter xylosoxidans* (*n* = 2) (0.5%) *Acinetobacter baumannii* (*n* = 20) (5.03%) *Burkholderia cepacia* (*n* = 1) (0.25%) *Myroides odoratimimus* (*n* = 4) (1%) *Pseudomonas aeruginosa* (*n* = 32) (8.06%) *Pseudomonas monteilii* (*n* = 1) (0.25%) *Stenotrophomonas maltophilia* (*n* = 2) (0.5%)

Percentages were calculated based on the total of 397 strains.

**Table 2:** Details of the isolates evaluated in the study, grouped by isolation site/sample.

No. of Isolates	No. of Isolates per Group	Isolation Site/Sample	Species
240	212 Enterobacterales 28 non-fermenter GNB	Urine	*Citrobacter braakii* (*n* = 1) *Citrobacter freundii* (*n* = 2) *Enterobacter cloacae* (*n* = 2) *Enterobacter hormaechei* (*n* = 1) *Escherichia coli* (*n* = 72) *Klebsiella aerogenes* (*n* = 2) *Klebsiella oxytoca* (*n* = 2) *Klebsiella pneumoniae* (*n* = 113) *Morganella morganii* (*n* = 3) *Proteus mirabilis* (*n* = 4) *Providencia rettgeri* (*n* = 2) *Providencia stuartii* (*n* = 6) *Serratia ureilytica* (*n* = 2) *Acinetobacter baumannii* (*n* = 4) *Myroides odoratimimus* (*n* = 4) *Pseudomonas aeruginosa* (*n* = 19) *Pseudomonas monteilii* (*n* = 1)
40	40 Enterobacterales	Nephrostomy urine	*Escherichia coli* (*n* = 3) *Klebsiella pneumoniae* (*n* = 21) *Morganella morganii* (*n* = 2) *Proteus mirabilis* (*n* = 2) *Providencia stuartii* (*n* = 1) *Providencia rettgeri* (*n* = 3) *Serratia marcescens* (*n* = 4) *Serratia ureilytica* (*n* = 4)
1	1 Enterobacterales	JJ stent urine	*Klebsiella pneumoniae* (*n* = 1)
1	1 Enterobacterales	Urine + blood culture	*Escherichia coli* (*n* = 1)
20	15 Enterobacterales 5 non-fermenter GNB	Blood/blood culture	*Enterobacter xiangfangensis* (*n* = 1) *Escherichia coli* (*n* = 1) *Klebsiella pneumoniae* (*n* = 12) *Providencia stuartii* (*n* = 1) *Achromobacter xylosoxidans* (*n* = 1) *Acinetobacter baumannii* (*n* = 1) *Pseudomonas aeruginosa* (*n* = 3)
9	9 Enterobacterales	Intravenous catheter	*Escherichia coli* (*n* = 1) *Klebsiella pneumoniae* (*n* = 7) *Proteus mirabilis* (*n* = 1)
35	21 Enterobacterales 14 non-fermenter GNB	Purulent/wound secretions	*Enterobacter cloacae* (*n* = 1) *Escherichia coli* (*n* = 5) *Klebsiella pneumoniae* (*n* = 11) *Morganella morganii* (*n* = 1) *Proteus mirabilis* (*n* = 1) *Providencia stuartii* (*n* = 1) *Serratia marcescens* (*n* = 1) *Acinetobacter baumannii* (*n* = 9) *Pseudomonas aeruginosa* (*n* = 5)
6	4 Enterobacterales 2 non-fermenter GNB	Pleural fluid	*Klebsiella pneumoniae* (*n =* 4) *Burkholderia cepacia* (*n* = 1) *Stenotrophomonas maltophilia* (*n* = 1)
32	19 Enterobacterales 13 non-fermenter GNB	Tracheal/bronchial secretions	*Enterobacter asburiae* (*n* = 1) *Enterobacter bugandensis* (*n* = 1) *Enterobacter cloacae* (*n* = 1) *Enterobacter kobei* (*n* = 1) *Klebsiella pneumoniae* (*n* = 11) *Proteus mirabilis* (*n* = 3) *Serratia marcescens* (*n* = 1) *Achromobacter xylosoxidans* (*n* = 1) *Acinetobacter baumannii* (*n* = 6) *Pseudomonas aeruginosa* (*n* = 5) *Stenotrophomonas maltophilia* (*n* = 1)
9	9 Enterobacterales	Sputum	*Enterobacter kobei* (*n* = 1) *Hafnia alvei* (*n* = 1) *Klebsiella pneumoniae* (*n* = 5) *Raoultella ornithinolytica* (*n* = 1) *Serratia marcescens* (*n* = 1)
2	2 Enterobacterales	Ascites fluid	*Klebsiella pneumoniae* (*n* = 2)
1	1 Enterobacterales	Bile	*Klebsiella oxytoca* (*n* = 1)
1	1 Enterobacterales	PEG tube	*Klebsiella pneumoniae* (*n* = 1)

Total number of strains *n* = 397.

**Table 3:** The phenotypes of the 45 *Klebsiella pneumoniae* strains resistant to all antimicrobials included in the disk diffusion panel.

No. of *K. pneumoniae* Strains	ESBL	Class A Carbapenemase (e.g., KPC)	Class B Carbapenemase (Metallo-Beta-Lactamase)	Class D Carbapenemase (e.g., OXA-48-Like)
1	−	+	−	−
1	+	−	−	+
1	−	−	+	−
1	−	+	+	−
9	−	−	+	+
18	+	−	+	+
14	+	−	+	−

**Table 4:** Cohen’s *κ* (kappa) inter-rater agreement calculated for the disk diffusion antibiogram evaluated for carbapenemase production and the 5 phenotypic tests performed for the detection of carbapenemase production in the 335 Enterobacterales strains.

	DD-AST CP	BCT	CIM	zCIM	mCIM	rCIM-A
DD-AST CP	1.00	[0.78–0.90]	[0.88–0.96]	[0.93–0.99]	[0.91–0.98]	[0.91–0.98]
BCT	0.84	1.00	[0.75–0.87]	[0.78–0.89]	[0.76–0.88]	[0.76–0.88]
CIM	0.92	0.81	1.00	[0.87–0.95]	[0.87–0.96]	[0.87–0.95]
zCIM	0.96	0.83	0.91	1.00	[0.96–1.00]	[0.93–0.99]
mCIM	0.94	0.82	0.92	0.98	1.00	[0.92–0.98]
rCIM-A	0.95	0.82	0.91	0.96	0.95	1.00

Values shown as *κ* (Cohen’s kappa), under the diagonal. The 95% confidence interval (CI) is shown in parentheses [x–y] above the diagonal. Cohen’s *κ* result: <0 no agreement; 0–0.20 slight (poor) agreement; 0.21–0.40 fair agreement; 0.41–0.60 moderate agreement; 0.61–0.80 substantial (good) agreement; 0.81–1 almost perfect (very good) agreement. *p* ≤ 0.01. DD-AST CP = disk diffusion antibiogram evaluated for carbapenemase production; BCT = BlueCarba Test; CIM = carbapenem inactivation method; zCIM = zinc CIM; mCIM = modified CIM; rCIM-A = rapid CIM-AmpC.

**Table 5:** Cohen’s *κ* (kappa) inter-rater agreement calculated for the 56 strains for which an NG-Test^®^ CARBA 5 was performed in relation to the disk diffusion antibiogram evaluated for carbapenemase production and the 5 phenotypic tests performed for the detection of carbapenemase-producing Enterobacterales.

	NG-Test^®^ CARBA 5	DD-AST CP	BCT	CIM	zCIM	mCIM	rCIM-A
NG-Test^®^ CARBA 5	1.00	[0.56–1.00]	[0.26–0.86]	[0.32–0.85]	[0.79–1.00]	[0.60–1.00]	[0.51–1.00]
DD-AST CP	0.79	1.00	[0.36–0.91]	[0.27–0.81]	[0.48–0.98]	[0.36–0.91]	[0.23–0.89]
BCT	0.56	0.64	1.00	[0.37–0.86]	[0.21–0.82]	[0.19–0.79]	[0.06–0.76]
CIM	0.58	0.54	0.62	1.00	[0.27–0.81]	[0.42–0.89]	[0.24–0.80]
zCIM	0.93	0.73	0.51	0.54	1.00	[0.71–1.00]	[0.43–0.98]
mCIM	0.81	0.63	0.49	0.66	0.87	1.00	[0.49–0.98]
rCIM-A	0.77	0.56	0.41	0.52	0.71	0.73	1.00

Values shown as *κ* (Cohen’s kappa) under the diagonal. The 95% CI is shown in parentheses [x–y] above the diagonal. Cohen’s *κ* result: <0 no agreement; 0–0.20 slight (poor) agreement; 0.21–0.40 fair agreement; 0.41–0.60 moderate agreement; 0.61–0.80 substantial (good) agreement; 0.81–1 almost perfect (very good) agreement. *p* ≤ 0.05. NG-Test^®^ CARBA 5: 48/56 strains positive for carbapenemase. DD-AST CP = disk diffusion antibiogram; BCT = BlueCarba Test; CIM = carbapenem inactivation method; zCIM = zinc CIM; mCIM = modified CIM; rCIM-A = rapid CIM-AmpC. Note: Despite very few differences, the kappa values are low, as they were subject to the kappa paradox. This happens when raters have high observed agreement but most data fall into one category, as Cohen’s kappa is sensitive to imbalances in outcome prevalence.

**Table 6:** Percentage agreement calculated for the 56 strains for which an NG-Test^®^ CARBA 5 was performed in relation to the disk diffusion antibiogram evaluated for carbapenemase production and the 5 phenotypic tests performed for the detection of carbapenemase-producing Enterobacterales.

	NG-Test^®^ CARBA 5	DD-AST CP	BCT	CIM	zCIM	mCIM	rCIM-A
NG-Test^®^ CARBA 5	100.00%						
DD-AST CP	98.04%	100.00%					
BCT	95.10%	97.06%	100.00%				
CIM	84.31%	86.27%	89.22%	100.00%			
zCIM	98.04%	100.00%	97.06%	86.27%	100.00%		
mCIM	94.12%	96.08%	99.02%	90.20%	96.08%	100.00%	
rCIM-A	98.04%	96.08%	93.14%	82.35%	96.08%	92.16%	100.00%

NG-Test^®^ CARBA 5: 48/56 strains positive for carbapenemase. DD-AST CP = disk diffusion antibiogram; BCT = BlueCarba Test; CIM = carbapenem inactivation method; zCIM = zinc CIM; mCIM = modified CIM; rCIM-A = rapid CIM-AmpC. Note: The percentage agreement metric has several limitations, as it does not account for agreement occurring by chance, can be influenced by low-prevalence categories, and does not penalize random guessing.

**Table 7:** The distribution of the 33 *Pseudomonas* spp. strains (32 *Pseudomonas aeruginosa* and 1 *Pseudomonas monteilii*) according to the colistin minimum inhibitory concentrations (MICs).

No. of *Pseudomonas* spp. Strains	Colistin MIC (μg/mL)
1	8
24 *	4
8	2

Negative control: *Escherichia coli* ATCC 25922 (MIC = 1 μg/mL). Positive control: *Escherichia coli* NCTC 13846 (*mcr*-1) (MIC = 8 μg/mL). * 1 of 24 strains was the *Pseudomonas monteilii* strain.

**Table 8:** The distribution of the 20 *Acinetobacter baumannii* strains according to the colistin minimum inhibitory concentrations (MICs).

No. of *Acinetobacter baumannii* Strains	Colistin MIC (μg/mL)
4	4
16	2

Negative control: *Escherichia coli* ATCC 25922 (MIC = 1 μg/mL). Positive control: *Escherichia coli* NCTC 13846 (*mcr*-1) (MIC = 8 μg/mL).

**Table 9:** Cohen’s *κ* (kappa) inter-rater agreement calculated for the carbapenemase detection lateral flow assays, the disk diffusion antibiogram evaluated for carbapenemase production, and the 5 phenotypic tests performed for the detection of carbapenemase-producing *Pseudomonas* spp. (*n* = 33) and *Acinetobacter baumannii* (*n* = 20).

	DD-AST CP	LF Assay	BCT	CIM	zCIM	mCIM	rCIM-A
DD-AST CP	1.00	1.00	[0.45–0.86]	[0.35–0.78]	[0.37–0.80]	[0.88–1.00]	1.00
LF Assay	1.00	1.00	[0.45–0.86]	[0.35–0.78]	[0.37–0.80]	[0.88–1.00]	1.00
BCT	0.66	0.66	1.00	[0.13–0.60]	[0.22–0.69]	[0.40–0.83]	[0.45–0.86]
CIM	0.57	0.57	0.36	1.00	[0.45–0.86]	[0.31–0.75]	[0.35–0.78]
zCIM	0.58	0.58	0.45	0.66	1.00	[0.32–0.77]	[0.37–0.80]
mCIM	0.96	0.96	0.61	0.53	0.55	1.00	[0.88–1.00]
rCIM-A	1.00	1.00	0.66	0.57	0.58	0.96	1.00

Values shown as *κ* (Cohen’s kappa) under the diagonal. The 95% CI is shown in parentheses [x–y] above the diagonal. Cohen’s *κ* result: <0 no agreement; 0–0.20 slight (poor) agreement; 0.21–0.40 fair agreement; 0.41–0.60 moderate agreement; 0.61–0.80 substantial (good) agreement; 0.81–1 almost perfect (very good) agreement. *p* ≤ 0.01. LF Assay = lateral flow (immunochromatographic) assay for carbapenemase detection; DD-AST CP = disk diffusion antibiogram; BCT = BlueCarba test; CIM = carbapenem inactivation method; zCIM = zinc CIM; mCIM = modified CIM; rCIM-A = rapid CIM-AmpC. Note: The low values observed for several assays are consistent with the known reduced sensitivity of these phenotypic tests for carbapenemase detection in non-fermenters compared to Enterobacterales [19,39,43].

## Data Availability

The original contributions presented in this study are included in the article/Supplementary Materials. Further inquiries can be directed to the corresponding authors.

## Supplementary Materials

The following supporting information can be downloaded at https://www.mdpi.com/article/10.3390/antibiotics15060580/s1, Table S1: Detailed results for the evaluated strains; Figure S1: Rough colony phenotype observed in 8 strains of *Klebsiella pneumoniae*; Figure S2: Colistin results obtained for 335 Enterobacterales strains using the disk-diffusion method, following older guidelines.

## References

[1] Murray C.J.L., Ikuta K.S., Sharara F., Swetschinski L., Robles Aguilar G., Gray A., Han C., Bisignano C., Rao P., Wool E. (2022). Global Burden of Bacterial Antimicrobial Resistance in 2019: A Systematic Analysis. Lancet.

[2] Centers for Disease Control and Prevention (2025). Antimicrobial Resistance: Causes and How It Spreads. https://www.cdc.gov/antimicrobial-resistance/causes/?CDC_AAref_Val=https://www.cdc.gov/drugresistance/where-resistance-spreads.html.

[3] Centers for Disease Control and Prevention (2024). Controlling the Emergence and Spread of Antimicrobial Resistance. https://www.cdc.gov/antimicrobial-resistance/prevention/?CDC_AAref_Val=https://www.cdc.gov/drugresistance/actions-to-fight.html.

[4] European Centre for Disease Prevention and Control (2017). ECDC Country Visit to Romania to Discuss Antimicrobial Issues. https://data.europa.eu/doi/10.2900/052263.

[5] Perestrelo S., Amaro A., Brouwer M.S.M., Clemente L., Ribeiro Duarte A.S., Kaesbohrer A., Karpíšková R., Lopez-Chavarrias V., Morris D., Prendergast D. (2023). Building an International One Health Strain Level Database to Characterise the Epidemiology of AMR Threats: ESBL—AmpC Producing *E. coli* as An Example—Challenges and Perspectives. Antibiotics.

[6] Bush K., Bradford P.A. (2020). Epidemiology of Β-Lactamase-Producing Pathogens. Clin. Microbiol. Rev..

[7] Jean S.-S., Harnod D., Hsueh P.-R. (2022). Global Threat of Carbapenem-Resistant Gram-Negative Bacteria. Front. Cell. Infect. Microbiol..

[8] Grundmann H., Glasner C., Albiger B., Aanensen D.M., Tomlinson C.T., Andrasević A.T., Cantón R., Carmeli Y., Friedrich A.W., Giske C.G. (2016). Occurrence of Carbapenemase-Producing Klebsiella Pneumoniae and *Escherichia coli* in the European Survey of Carbapenemase-Producing Enterobacteriaceae (EuSCAPE): A Prospective, Multinational Study. Lancet Infect. Dis..

[9] Choi J.-H., Ali M.S., Moon B.-Y., Kang H.-Y., Kim S.-J., Song H.-J., Mechesso A.F., Moon D.-C., Lim S.-K. (2023). Prevalence and Characterization of Extended-Spectrum β-Lactamase-Producing *Escherichia coli* Isolated from Dogs and Cats in South Korea. Antibiotics.

[10] Colosi I.A., Baciu A.M., Opriș R.V., Peca L., Gudat T., Simon L.M., Colosi H.A., Costache C. (2020). Prevalence of ESBL, AmpC and Carbapenemase-Producing Enterobacterales Isolated from Raw Vegetables Retailed in Romania. Foods.

[11] Timofte D., Maciuca I.E., Williams N.J., Wattret A., Schmidt V. (2016). Veterinary Hospital Dissemination of CTX-M-15 Extended-Spectrum Beta-Lactamase–Producing *Escherichia coli* ST410 in the United Kingdom. Microb. Drug Resist..

[12] Lixandru B.E., Cotar A.I., Straut M., Usein C.R., Cristea D., Ciontea S., Tatu-Chitoiu D., Codita I., Rafila A., Nica M. (2015). Carbapenemase-Producing *Klebsiella pneumoniae* in Romania: A Six-Month Survey. PLoS ONE.

[13] Popescu G.A., Codiță I., Szekely E., Șerban R., Ruja G., Tălăpan D. (2016). Ghid privind *Enterobacteriaceae* Producătoare de Carbapenemaze: Diagnosticul, Prevenirea Transmiterii Interumane și Tratamentul Infecțiilor Produse. https://insp.gov.ro/wpfb-file/ghid-privind-enterobacteriaceae-producatoare-de-carbapenemaze-diagnosticul-prevenirea-transmiterii-interumane-si-tratamentul-infectiilor-produse-pdf/.

[14] Molnár S., Vas K.E., Székely E. (2020). Carbapenemase Producing Enterobacterales in Romania: Investigating the Origins. Rev. Română Med. Lab..

[15] Resman F. (2020). Antimicrobial Stewardship Programs; a Two-Part Narrative Review of Step-Wise Design and Issues of Controversy Part I: Step-Wise Design of an Antimicrobial Stewardship Program. Ther. Adv. Infect. Dis..

[16] Bizo P.T., Dumitras D., Popa A. (2015). Evaluation of Restricted Antibiotic Use in a Hospital in Romania. Int. J. Clin. Pharm..

[17] Pirii L.E., Friedrich A.W., Rossen J.W.A., Vogels W., Beerthuizen G.I.J.M., Nieuwenhuis M.K., Kooistra-Smid A.M.D., Bathoorn E. (2017). Extensive Colonization with Carbapenemase-Producing Microorganisms in Romanian Burn Patients: Infectious Consequences from the Colectiv Fire Disaster. Eur. J. Clin. Microbiol. Infect. Dis..

[18] Institutul Național de Boli Infecțioase “Prof. Dr. Matei Balș” din București (2021). Ghid pentru Prevenirea și Limitarea Fenomenului de Rezistență la Antimicrobiene (AMR) și a Infecțiilor Asociate Asistenței Medicale (IAAM). https://www.mateibals.ro/index.php/cercetare-si-proiecte/pdp-8-proiect-amr.

[19] The European Committee on Antimicrobial Susceptibility Testing (2017). EUCAST Guidelines for Detection of Resistance Mechanisms and Specific Resistances of Clinical and/or Epidemiological Importance. Version 2.0. https://www.eucast.org/fileadmin/src/media/PDFs/EUCAST_files/Resistance_mechanisms/EUCAST_detection_of_resistance_mechanisms_170711.pdf.

[20] Sepp E., Andreson R., Balode A., Bilozor A., Brauer A., Egorova S., Huik K., Ivanova M., Kaftyreva L., Kõljalg S. (2019). Phenotypic and Molecular Epidemiology of ESBL-, AmpC-, and Carbapenemase-Producing *Escherichia coli* in Northern and Eastern Europe. Front. Microbiol..

[21] Hammoudi Halat D., Ayoub Moubareck C. (2020). The Current Burden of Carbapenemases: Review of Significant Properties and Dissemination among Gram-Negative Bacteria. Antibiotics.

[22] Jacoby G.A. (2009). AmpC β-Lactamases. Clin. Microbiol. Rev..

[23] Tamma P.D., Doi Y., Bonomo R.A., Johnson J.K., Simner P.J., Group A.R.L., Tamma P.D., Doi Y., Bonomo R.A. (2019). A Primer on AMPC Β-Lactamases: Necessary Knowledge for an Increasingly Multidrug-Resistant World. Clin. Infect. Dis..

[24] Thomson K.S. (2010). Extended-Spectrum-Β-Lactamase, AMPC, and Carbapenemase Issues. J. Clin. Microbiol..

[25] David S., Cohen V., Reuter S., Sheppard A.E., Giani T., Parkhill J., Rossolini G.M., Feil E.J., European Survey of Carbapenemase-Producing Enterobacteriaceae (EuSCAPE) Working Group, ESCMID Study Group for Epidemiological Markers (ESGEM) (2020). Integrated Chromosomal and Plasmid Sequence Analyses Reveal Diverse Modes of Carbapenemase Gene Spread among *Klebsiella pneumoniae*. Proc. Natl. Acad. Sci. USA.

[26] Beshah D., Desta A.F., Woldemichael G.B., Belachew E.B., Derese S.G., Zelelie T.Z., Desalegn Z., Tessema T.S., Gebreselasie S., Abebe T. (2023). High Burden of ESBL and Carbapenemase-Producing Gram-Negative Bacteria in Bloodstream Infection Patients at a Tertiary Care Hospital in Addis Ababa, Ethiopia. PLoS ONE.

[27] Drawz S.M., Bonomo R.A. (2010). Three Decades of Β-Lactamase Inhibitors. Clin. Microbiol. Rev..

[28] León-Sampedro R., DelaFuente J., Díaz-Agero C., Crellen T., Musicha P., Rodríguez-Beltrán J., De La Vega C., Hernández-García M., Group R.-G.W.S., López-Fresneña N. (2021). Pervasive Transmission of a Carbapenem Resistance Plasmid in the Gut Microbiota of Hospitalized Patients. Nat. Microbiol..

[29] White L., Hopkins K.L., Meunier D., Perry C.L., Pike R., Wilkinson P., Pickup R.W., Cheesbrough J., Woodford N. (2016). Carbapenemase-Producing *Enterobacteriaceae* in Hospital Wastewater: A Reservoir That May Be Unrelated to Clinical Isolates. J. Hosp. Infect..

[30] Nordmann P., Poirel L. (2019). Epidemiology and Diagnostics of Carbapenem Resistance in Gram-Negative Bacteria. Clin. Infect. Dis..

[31] Pascual Á., Pintado V., Rodríguez-Baño J., Miró J.M. (2014). Carbapenemase-Producing *Enterobacteriaceae*: The End of the Antibiotic Era?. Enfermedades Infecc. Microbiol. Clínica.

[32] Bonnin R.A., Jousset A.B., Emeraud C., Oueslati S., Dortet L., Naas T. (2021). Genetic Diversity, Biochemical Properties, and Detection Methods of Minor Carbapenemases in Enterobacterales. Front. Med..

[33] Papp-Wallace K.M., Endimiani A., Taracila M.A., Bonomo R.A. (2011). Carbapenems: Past, Present, and Future. Antimicrob. Agents Chemother..

[34] Rus M., Licker M., Musuroi C., Seclaman E., Muntean D., Cirlea N., Tamas A., Vulpie S., Horhat F.G., Baditoiu L. (2020). Distribution of NDM1 Carbapenemase-Producing *Proteeae* Strains on High-Risk Hospital Wards. Infect. Drug Resist..

[35] Molnár S., Flonta M.M.M., Almaş A., Buzea M., Licker M., Rus M., Földes A., Székely E. (2019). Dissemination of NDM-1 Carbapenemase-Producer *Providencia stuartii* Strains in Romanian Hospitals: A Multicentre Study. J. Hosp. Infect..

[36] Coyne A.J.K., Ghali A.E., Holger D., Rebold N., Rybak M.J. (2022). Therapeutic Strategies for Emerging Multidrug-Resistant *Pseudomonas aeruginosa*. Infect. Dis. Ther..

[37] Oliva A., Mascellino M.T., Cipolla A., D’Abramo A., De Rosa A., Savinelli S., Ciardi M.R., Mastroianni C.M., Vullo V. (2015). Therapeutic Strategy for Pandrug-Resistant *Klebsiella pneumoniae* Severe Infections: Short-Course Treatment with Colistin Increases the in Vivo and in Vitro Activity of Double Carbapenem Regimen. Int. J. Infect. Dis..

[38] Magiorakos A.-P., Srinivasan A., Carey R.B., Carmeli Y., Falagas M.E., Giske C.G., Harbarth S., Hindler J.F., Kahlmeter G., Olsson-Liljequist B. (2011). Multidrug-Resistant, Extensively Drug-Resistant and Pandrug-Resistant Bacteria: An International Expert Proposal for Interim Standard Definitions for Acquired Resistance. Clin. Microbiol. Infect..

[39] Humphries R.M. (2019). CIM City: The Game Continues for a Better Carbapenemase Test. J. Clin. Microbiol..

[40] Rahmani S., Basu S., Simner P.J., Kambli P., Shetty A., Rodrigues C. (2025). Evaluation of the Rapid Lateral Flow Assay (LFA) for Detection of Five Major Carbapenemase Enzyme Families in Genotypically Characterised Bacterial Isolates. Indian J. Med. Res..

[41] Tarlton N.J., Wallace M.A., Potter R.F., Zhang K., Dantas G., Dubberke E.R., Burnham C.-A.D., Yarbrough M.L. (2023). Evaluation of the NG-Test CARBA 5 Lateral Flow Assay with an IMP-27-Producing *Morganella morganii* and Other *Morganellaceae*. Microbiol. Spectr..

[42] Moreira N.K., Wilhelm C.M., Wink P.L., Barth A.L., Caierão J. (2022). MALDI-TOF Mass Spectrometry for Direct KPC Detection among Enterobacterales. Braz. J. Microbiol..

[43] Sattler J., Brunke A., Hamprecht A. (2021). Systematic Comparison of Three Commercially Available Combination Disc Tests and the Zinc-Supplemented Carbapenem Inactivation Method (ZCIM) for Carbapenemase Detection in Enterobacterales Isolates. J. Clin. Microbiol..

[44] Muntean M.-M., Muntean A.-A., Gauthier L., Creton E., Cotellon G., Popa M.I., Bonnin R.A., Naas T. (2018). Evaluation of the Rapid Carbapenem Inactivation Method (rCIM): A Phenotypic Screening Test for Carbapenemase-Producing *Enterobacteriaceae*. J. Antimicrob. Chemother..

[45] Muntean M.M., Muntean A.-A., Guerin F., Cattoir V., Creton E., Cotellon G., Oueslati S., Popa M.I., Girlich D., Iorga B.I. (2021). Optimization of the Rapid Carbapenem Inactivation Method for Use with AmpC Hyperproducers. J. Antimicrob. Chemother..

[46] Muntean A.-A., Poenaru A., Neagu A., Caracoti C., Muntean M.M., Popa V.T., Bogdan M.A., Naas T., Popa M.I. (2018). Use of the Rapid Carbapenem Inactivation Method (rCIM) with Carbapenemase Inhibitors: A Proof of Concept Experiment. Roum. Arch. Microbiol. Immunol..

[47] Ding L., Shen S., Chen J., Tian Z., Shi Q., Han R., Guo Y., Hu F. (2023). *Klebsiella pneumoniae* Carbapenemase Variants: The New Threat to Global Public Health. Clin. Microbiol. Rev..

[48] Wyres K.L., Lam M.M.C., Holt K.E. (2020). Population Genomics of *Klebsiella pneumoniae*. Nat. Rev. Microbiol..

[49] Galani I., Kontopidou F., Souli M., Rekatsina P.-D., Koratzanis E., Deliolanis J., Giamarellou H. (2008). Colistin Susceptibility Testing by Etest and Disk Diffusion Methods. Int. J. Antimicrob. Agents.

[50] Qin J., Zhu Y., Zhu Y., Gao Q., Zhang H., Li M., Shen Z. (2024). Emergence of Silent NDM-1 Carbapenemase Gene in Carbapenem-Susceptible Klebsiella Pneumoniae: Clinical Implications and Epidemiological Insights. Drug Resist. Updates.

[51] Emira A.S., Madkour L.A.E.-F., Seif N.E., Dwedar R.A. (2020). Expressed and Silent Carbapenemase Genes Detected by Multiplex PCR in Both Carbapenem-Resistant and Phenotypically-Susceptible Gram Negative Bacilli. Alex. J. Med..

[52] Álvarez V.E., Knecht C., Piekar M., Allende N.G., Machuca A.G., Campos J., Carpio E., Páez L., Fox B., Canigia L.F. (2025). Genomic Characterization Reveals Infections and Silent Dissemination of Carbapenemase-Producing Klebsiella Oxytoca Complex Associated with ST2 Lineage in a Hospital Setting. Curr. Microbiol..

[53] Société Française de Microbiologie, Comité de l’Antibiogramme de la Société Française de Microbiologie (2025). Recommandations 2025 V.1.1 Juillet. https://www.sfm-microbiologie.org/wp-content/uploads/2025/07/CASFM2025_V1.1-JUILLET-2025.pdf.

[54] Perry J.D., Butterworth L.A., Nicholson A., Appleby M.R., Orr K.E. (2003). Evaluation of a New Chromogenic Medium, Uriselect 4, for the Isolation and Identification of Urinary Tract Pathogens. J. Clin. Pathol..

[55] Muntean A.-A., Muntean M.-M., Hogea M.-O., Chelaru E.-C., Popa M.I. (2023). Antibiogram Picture Guide for the Medical Microbiology Practitioner—Part 2: Common Carbapenemases in Enterobacterales. Roum. Arch. Microbiol. Immunol..

[56] Muntean A.-A., Muntean M.-M., Popa G.L. (2021). Antibiogram Picture Guide for the Medical Microbiology Practitioner—Part 1: Extended-Spectrum Beta-Lactamases (ESBLs) and Cephalosporinases (AmpCs) in Enterobacterales. Roum. Arch. Microbiol. Immunol..

[57] The European Committee on Antimicrobial Susceptibility Testing. Clinical Breakpoints and Dosing of Antibiotics. https://www.eucast.org/clinical_breakpoints.

[58] The European Committee on Antimicrobial Susceptibility Testing. Disk Diffusion and Quality Control. https://www.eucast.org/bacteria/methodology-and-instructions/disk-diffusion-and-quality-control/.

[59] Chelaru E.-C., Muntean A.-A., Muntean M.-M., Hogea M.-O., Caracoti C.-Ș., Ciomaga B.-F., Naas T., Popa M.I. (2025). Oxacillin-Supplemented Mueller-Hinton Agar for In Vitro Inhibition of Ambler Class C β-Lactamases in Enterobacterales. Antibiotics.

[60] Fournier D., Garnier P., Jeannot K., Mille A., Gomez A.-S., Plésiat P. (2013). A Convenient Method To Screen for Carbapenemase-Producing *Pseudomonas aeruginosa*. J. Clin. Microbiol..

[61] CNR Résistance aux Antibiotiques (2017). Detection des Beta-Lactamases a Spectre Etendu de Classe a et d par la Realisation de Tests de Synergie Chez *Pseudomonas aeruginosa*, Fiche d’Informations. https://www.cnr-resistance-antibiotiques.fr/ressources/pages/Tests_synergie_BLSE_V1.pdf.

[62] Jeannot K., Fournier D., Müller E., Cholley P., Plésiat P. (2013). Clonal Dissemination of *Pseudomonas aeruginosa* Isolates Producing Extended-Spectrum β-Lactamase SHV-2a. J. Clin. Microbiol..

[63] Fournier D., Grisot E., Potron A., Liapis E., Jeannot K., Plésiat P. Nouvelle Stratégie de Dépistage des *P. aeruginosa* BLSE ou Carbapénèmase Positifs. https://www.cnr-resistance-antibiotiques.fr/ressources/pages/Poster_RICAI_CT_algorithmev2.pdf.

[64] CNR Résistance aux Antibiotiques (2024). Etude des Mécanismes de Résistance Chez *A. baumannii*: Logigramme. Version 5. https://www.cnr-resistance-antibiotiques.fr/ressources/pages/v5_logigramme_prise_en_charge_Acinetobacter.pdf.

[65] Poirel L., Menuteau O., Agoli N., Cattoen C., Nordmann P. (2003). Outbreak of Extended-Spectrum β-Lactamase VEB-1-Producing Isolates of *Acinetobacter baumannii* in a French Hospital. J. Clin. Microbiol..

[66] Bhowmick T., Canton R., Pea F., Quevedo J., Henriksen A.S., Timsit J.-F., Kaye K.S. (2025). Cefepime-Enmetazobactam: First Approved Cefepime-β-Lactamase Inhibitor Combination for Multi-Drug Resistant Enterobacterales. Future Microbiol..

[67] Kohlmann R., Bähr T., Gatermann S.G. (2019). Effect of AmpC Derepression on Cefepime MIC in Enterobacterales with Chromosomally Encoded Inducible AmpC β-Lactamase. Clin. Microbiol. Infect..

[68] Tamma P.D., Girdwood S.C.T., Gopaul R., Tekle T., Roberts A.A., Harris A.D., Cosgrove S.E., Carroll K.C. (2013). The Use of Cefepime for Treating AMPC Β-Lactamase–Producing *Enterobacteriaceae*. Clin. Infect. Dis..

[69] Laborda P., Colque C.A., La Rosa R., Molin S., Johansen H.K. (2025). Carbapenem-Resistance oprD Mutations Reshape Pseudomonas Aeruginosa Host-Pathogen Interactions during Infection. Nat. Commun..

[70] Pires J., Novais Â., Peixe L. (2013). Blue-Carba, an Easy Biochemical Test for Detection of Diverse Carbapenemase Producers Directly from Bacterial Cultures. J. Clin. Microbiol..

[71] García-Fernández S., Morosini M.-I., Gijón D., Beatobe L., Ruiz-Garbajosa P., Domínguez L., Cantón R., Valverde A. (2015). Detection of Carbapenemase Production in a Collection of *Enterobacteriaceae* with Characterized Resistance Mechanisms from Clinical and Environmental Origins by Use of Both Carba NP and Blue-Carba Tests. J. Clin. Microbiol..

[72] Van Der Zwaluw K., De Haan A., Pluister G.N., Bootsma H.J., De Neeling A.J., Schouls L.M. (2015). The Carbapenem Inactivation Method (CIM), a Simple and Low-Cost Alternative for the CARBA NP Test to Assess Phenotypic Carbapenemase Activity in Gram-Negative Rods. PLoS ONE.

[73] Pierce V.M., Simner P.J., Lonsway D.R., Roe-Carpenter D.E., Johnson J.K., Brasso W.B., Bobenchik A.M., Lockett Z.C., Charnot-Katsikas A., Ferraro M.J. (2017). Modified Carbapenem Inactivation Method for Phenotypic Detection of Carbapenemase Production among *Enterobacteriaceae*. J. Clin. Microbiol..

[74] Simner P.J., Johnson J.K., Brasso W.B., Anderson K., Lonsway D.R., Pierce V.M., Bobenchik A.M., Lockett Z.C., Charnot-Katsikas A., Westblade L.F. (2017). Multicenter Evaluation of the Modified Carbapenem Inactivation Method and the Carba NP for Detection of Carbapenemase-Producing *Pseudomonas aeruginosa* and *Acinetobacter baumannii*. J. Clin. Microbiol..

[75] Howard J.C., Creighton J., Ikram R., Werno A.M. (2020). Comparison of the Performance of Three Variations of the Carbapenem Inactivation Method (CIM, Modified CIM [mCIM] and in-House Method (iCIM)) for the Detection of Carbapenemase-Producing Enterobacterales and Non-Fermenters. J. Glob. Antimicrob. Resist..

[76] Singh S., Basu S., Mirza S., Karyakarte R., Rodrigues C., Bergman Y., Naik M., Randive B., Jacobs E., Gupta A. (2025). Variability of Reagents Matters—Enhancements to the CLSI Modified Carbapenem Inactivation Method Outside the United States to Improve Accuracy. J. Clin. Microbiol..

