# Supplementary material for: Evaluation of Beta-Lactamase-Producing Strains Isolated in a Tertiary Nephrology Hospital in Romania
Source: Antibiotics (Basel). 2026 Jun 7;15(6):580. doi: 10.3390/antibiotics15060580 (PMC13295298; doi:10.3390/antibiotics15060580)
Supplement: Supplementary file 1 [file antibiotics-15-00580-s001.zip › Supplementary figures.pdf]

antibiotics

MDPI

Article

Evaluation of Beta-Lactamase-Producing Strains Isolated in a Tertiary Nephrology Hospital in Romania

Edgar-Costin Chelaru [1,2,*,†], Andrei-Alexandru Muntean [1,2,†], Ioana Manea [1,3], Mihai-Octav Hogea [1], Crina-Mihaela Dinuță [1], Mioara Mazăre [1], Mădălina-Maria Muntean [1], Călin-Constantin Ghițulescu [1], Bogdan-Florin Ciomaga [1], Costin-Ștefan Caracoti [1,2], Diana-Maria Preoteasa [1] and Mircea Ioan Popa [1,2,*]

[1] Discipline of Microbiology II, Department 2, Faculty of Medicine, Carol Davila University of Medicine and Pharmacy, 020021 Bucharest, Romania
[2] Cantacuzino National Military Medical Institute for Research and Development, 050096 Bucharest, Romania
[3] Dr. Carol Davila Clinical Nephrology Hospital, 010731 Bucharest, Romania
* Correspondence: edgar-costin.chelaru@drd.umfcd.ro (E.-C.C.); mircea.ioan.popa@umfcd.ro (M.I.P.)
† These authors contributed equally to this work.

Supplementary figures

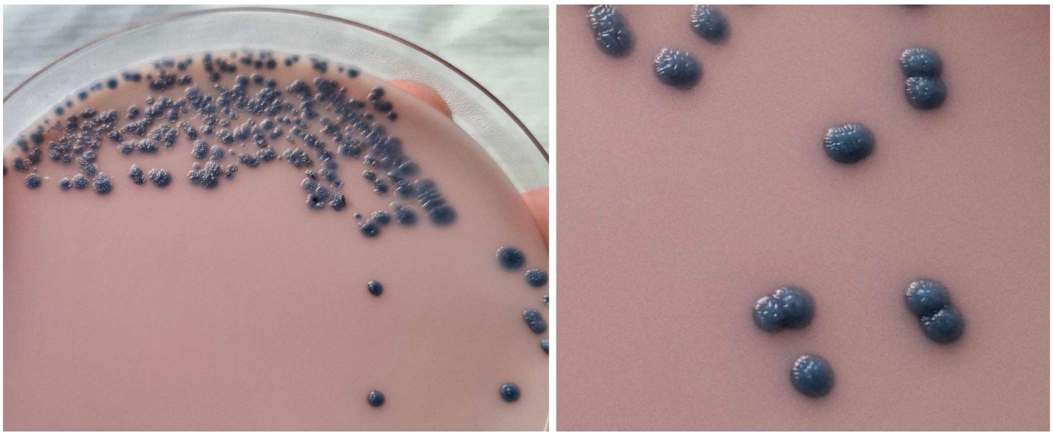

Figure S1. Rough colony phenotype observed in 8 strains of *Klebsiella pneumoniae*.

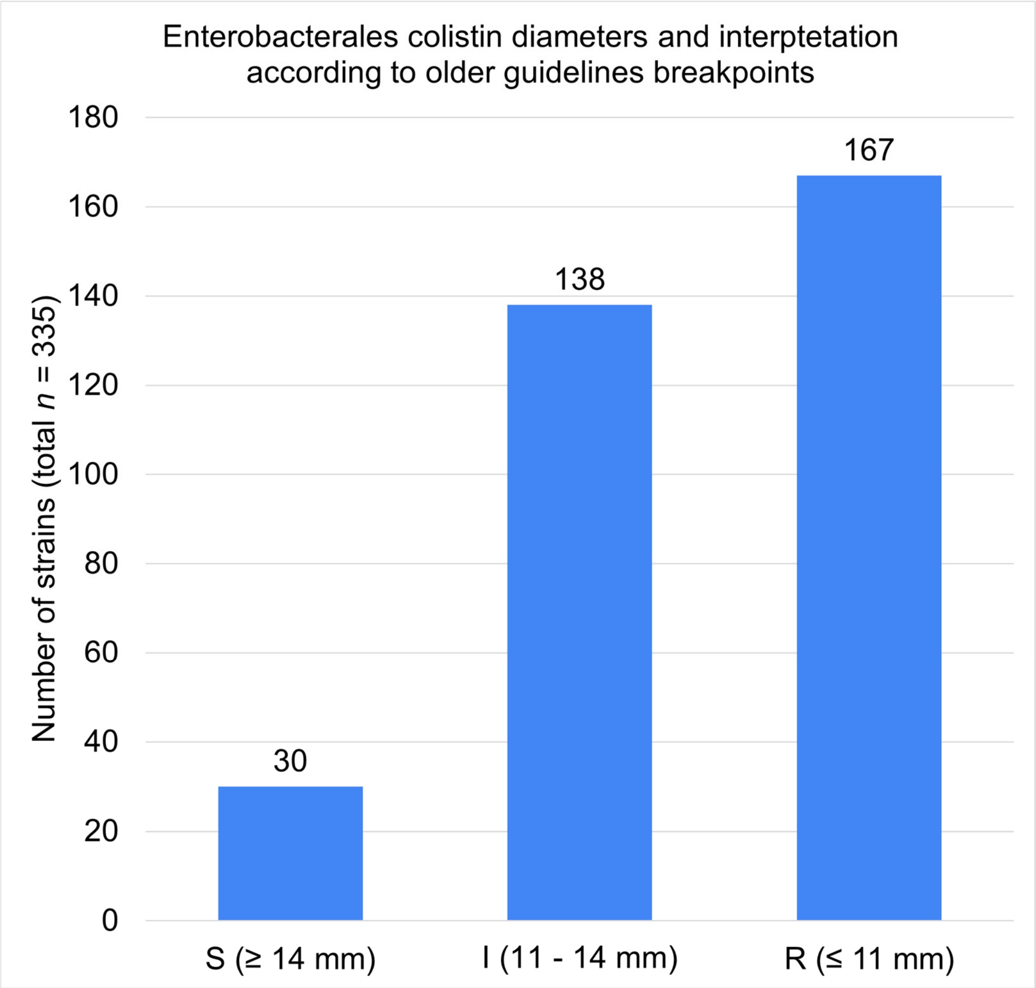

Figure S2. Colistin results obtained for 335 Enterobacterales strains using the disk-diffusion method, following older guidelines. S = susceptible; I = intermediate; R = resistant. Note: Results are presented for historical and exploratory reference only and should not be used for clinical decision-making.
